# Solid‐State NMR‐Assisted Dynamic Characterization of two Isostructural Solvates of 5α‐Bromo‐6β,19‐Epoxy‐Androstan‐3β,17β‐Diol Diacetate

**DOI:** 10.1002/mrc.5517

**Published:** 2025-02-18

**Authors:** Josué Vazquez‐Chavez, Armando Navarro‐Huerta, Marcos Flores‐Álamo, Braulio Rodríguez‐Molina, Martín A. Iglesias‐Arteaga

**Affiliations:** ^1^ Facultad de Química Universidad Nacional Autónoma de México Mexico City Mexico; ^2^ Instituto de Química, Universidad Nacional Autónoma de México Mexico City Mexico

**Keywords:** ^13^C CPMAS, ^2^H Quadrupolar resonance, Arrhenius, crystalline material, Eyring, solid‐state NMR, solvate, steroid

## Abstract

The study reports on the rotational dynamics of acetone‐*d*_6_ (Form **II**) and DMSO‐*d*_6_ (Form **III**) within the crystalline structures of two solvates of 5α‐bromo‐6β,19‐epoxy‐androstan‐3β,17β‐diol diacetate (Compound **1**) by means of solid‐state Nuclear Magnetic Resonance through the quadrupolar ^2^H spin‐echo technique. The spectral data allowed the determination of the activation barriers (*E*_a_) for rotation of the solvent molecules, with 6.24 kcal mol^−1^ for deuterated acetone in the Form **II**‐*d*_*6*_ and 8.19 kcal mol^−1^ for the case of deuterated dimethylsulfoxide in Form **III**‐*d*_*6*_. The use of calculations and the Transition State theory through the linear Eyring equation suggested that although the acetone molecules experience a low activation energy (*E*_*a*_ = 6.24 kcal mol^−1^), a highly ordered transition state during the molecular motion reduces its rotational rate. Conversely, the DMSO molecules, with a higher activation barrier (*E*_*a*_ = 8.19 kcal mol^−1^) attributed to a denser packing coefficient, have faster motional rates. Based on complementary X‐ray and NMR spectroscopy techniques, this work provides detailed insights into the mechanistic phenomena involved in the mobility of small molecules inside crystalline arrangements.

## Graphical abstract

The crystallization of the compound 5α‐bromo‐6β,19‐epoxy‐androstan‐3β,17β‐diol diacetate (**1**) from saturated solutions of acetone and DMSO yielded two isostructural solvates (Forms **II** and **III**, respectively). Using microcrystalline samples of the solvates and solid‐state NMR, through the ^2^H Quadrupolar Spin‐echo pulse sequence, it was determined the activation barriers for rotation (*E*_*a*_) of deuterated solvent molecules in the solvated forms.

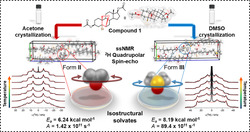

## 1 Introduction

Steroids constitute a vast and essential family of polycyclic compounds. A wide variety of members of this family are involved in biochemical processes that guarantee the reproduction, development, and maintenance of life of organisms of both the animal and vegetable kingdoms [1]. In addition, steroid‐based treatments for important diseases [2] and birth control [3] have positioned these compounds in a paramount place within the pharmaceutical industry. All this has kept steroids in the spotlight of scientists for more than a century. The last four decades have witnessed the implementation of steroid applications in the field of crystalline materials. Several crystalline steroid‐based molecular rotors, [4, 5, 6] porous solids [7, 8, 9], and inclusion complexes [10] have been reported.

The multiple steroid applications have fueled the development of synthetic tools for the introduction of structural modifications. In particular, the functionalization of angular C‐19 methyl group attached to position C‐10 has attracted considerable attention and can be achieved by the generation of 6β,19‐epoxy‐steroids. These bridged compounds are the most reliable intermediates to access C‐19 oxygenated bioactive steroids [11, 12, 13, 14, 15], or C‐19 functionalized biorthogonal probes for imaging in cells [16], and C‐19 bridged fluorescent steroid dimer [17].

**SCHEME 1:**
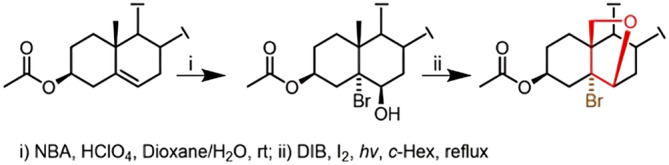
Typical synthesis of 5α‐bromo‐6β,19‐epoxy‐steroids.

A typical sequence of the preparation of 5α‐bromo‐6β,19‐epoxy‐steroids comprises the generation of 5α‐bromo‐6β‐hydroxysteroids that on photolysis in the presence diacetoxyiodobenzene (DIB) and iodine leads to the desired compounds (Scheme 1) [18].

Additionally, the shape and chirality of steroids provide an exciting starting point for applications in the solid state, given their availability and biological importance. It is known that steroids are prone to display *solvatomorphism* [19, 20, 21], a specific type of polymorphism dictated by the recrystallization solvent, changing the stability of the resulting crystalline form. Although this phenomenon has been documented in several reports [22], the solvent itself may display a particular dynamic behavior inside the crystal structures. For instance, THF molecules show a preferential axis of rotation within carbazole‐based solvate cocrystals, thanks to specific intermolecular interactions [23]. Thus, the dynamics of solvent molecules in steroidal crystal forms is still a vast area for exploration. The modulation of internal dynamics is a long‐sought feature in crystalline materials, owing to the influence of the molecular motions in several properties, such as radiative relaxation pathways [24]. Acetone and DMSO are two of the most common solvents for studying the dynamic processes of solvent molecules. Their chemical similarity and comparison with other carbonyl compounds and their capabilities to form hydrogen bonds through the *sp* ^*2*^ oxygen make them ideal probes for unveiling rotational dynamic processes [25, 26].

**FIGURE 1:**
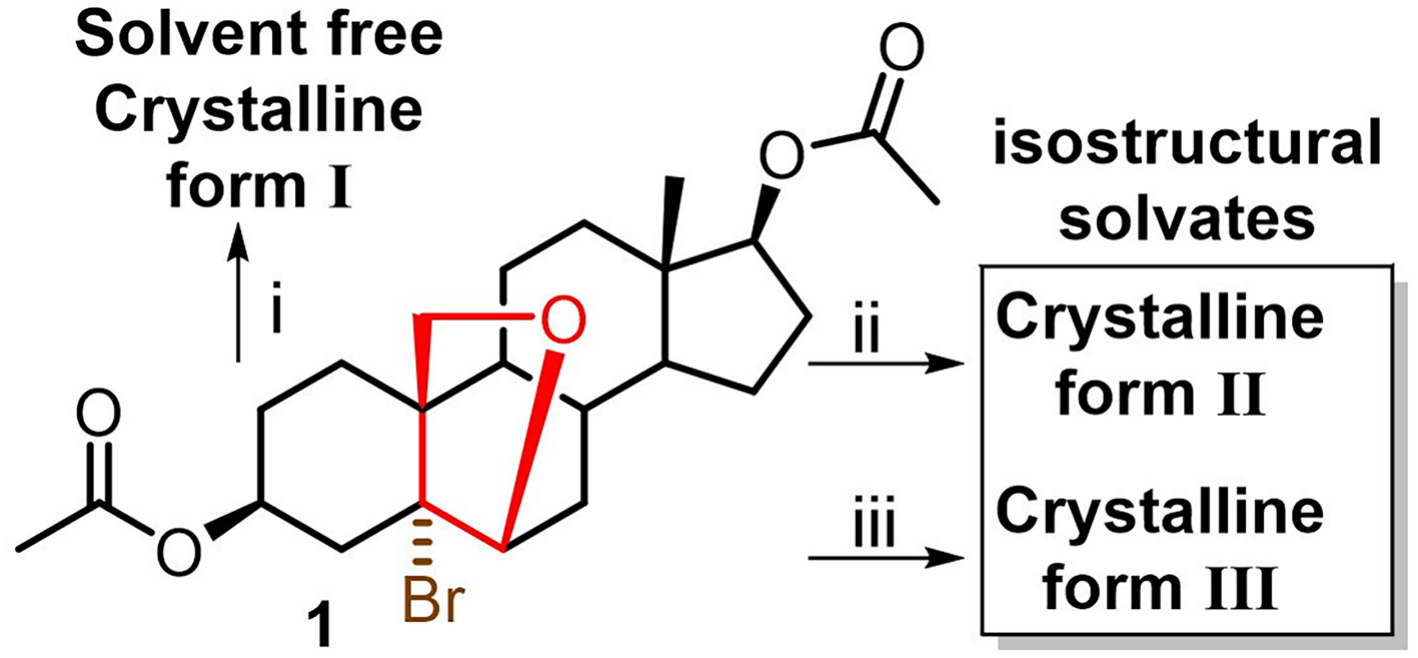
Structure of the title compound and outcome of crystallization experiments.

During our investigations, we found that the title compound 5α‐bromo‐6β,19‐epoxy‐androstan‐3β,17β‐diol diacetate (**1**) is prone to crystallize from almost every solvent or solvent mixture that was dissolved into. Interestingly, while using methanol, dichloromethane/hexane, dioxane/water, and methyl acetate/hexane mixtures (**Condition i**), the crystallization procedure yielded the same solvent‐free form. However, the crystallization in acetone (**Condition ii**) or dimethylsulfoxide (**Condition iii**) produced two isostructural solvates (Figure 1). The inspection of the three X‐ray structures (solvent‐free Form **I** and two solvated Forms **II** and **III**) prompted us to characterize each form and investigate the dynamic processes of the occluded solvent molecules.

Herein, we describe the use of the solid‐state nuclear magnetic resonance quadrupolar ^2^H spin‐echo technique to unveil details on the phenomena and rotational barriers involved in the dynamics processes of small solvent molecules like acetone or dimethyl sulfoxide inside the cavities of the crystalline arrangements of 5α‐bromo‐6β,19‐epoxy‐androstan‐3β,17β‐diol diacetate (**1**).

## 2 Results and Discussion

### 2.1 Crystallographic Features of Compound 1

**FIGURE 2:**
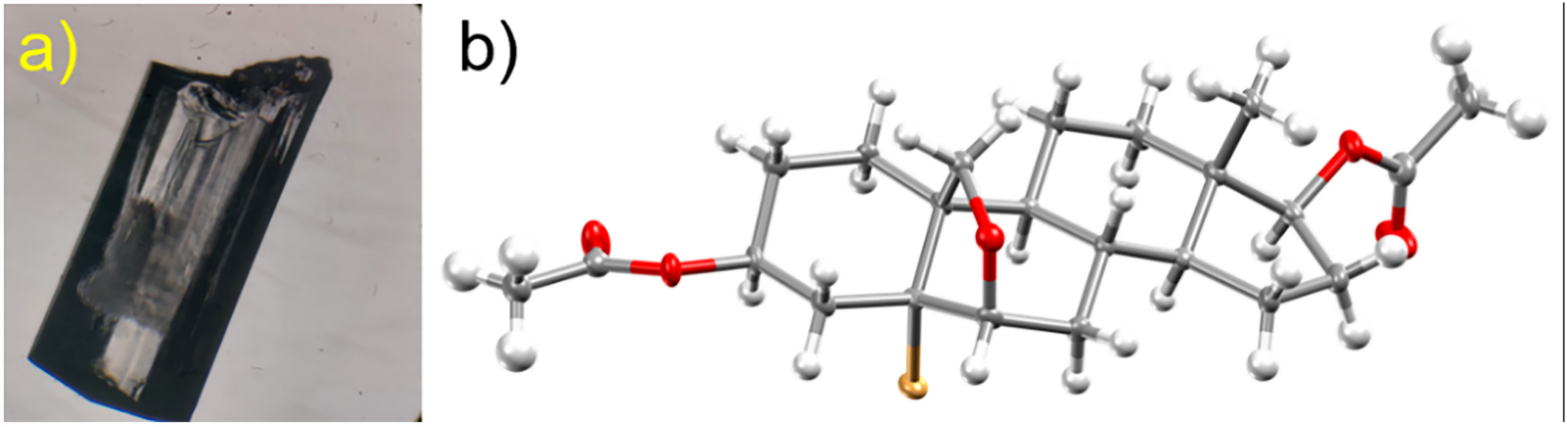
(a) Microscope image of prismatic‐shaped crystals of **1** (Form **I**) and (b) one of the two molecules of **1** in the asymmetric unit of Form **I**.

Compound **1** was synthesized using a modification of the synthetic sequence illustrated in Scheme 1 (see the Supporting Information for details) and crystallized using several mixtures of solvents. Interestingly, in dichloromethane/hexane, dioxane/water, methanol, and methyl acetate/hexane mixtures, Compound **1** crystallizes as solvent‐free (Form **I**) with prismatic habits, as seen in Figure 2a. The SC‐XRD data collections at 130 K for this structure revealed an orthorhombic system with a noncentrosymmetric *P*2_1_2_1_2_1_ space group. The cell constants for the different crystals obtained are consistent among them, with variations no larger than 0.1 Å, as visualized in Table S1. The asymmetric unit of this crystalline form comprises two molecules of **1** (Figure 2b), whereas eight molecules are embedded into the unit cell.

**FIGURE 3:**
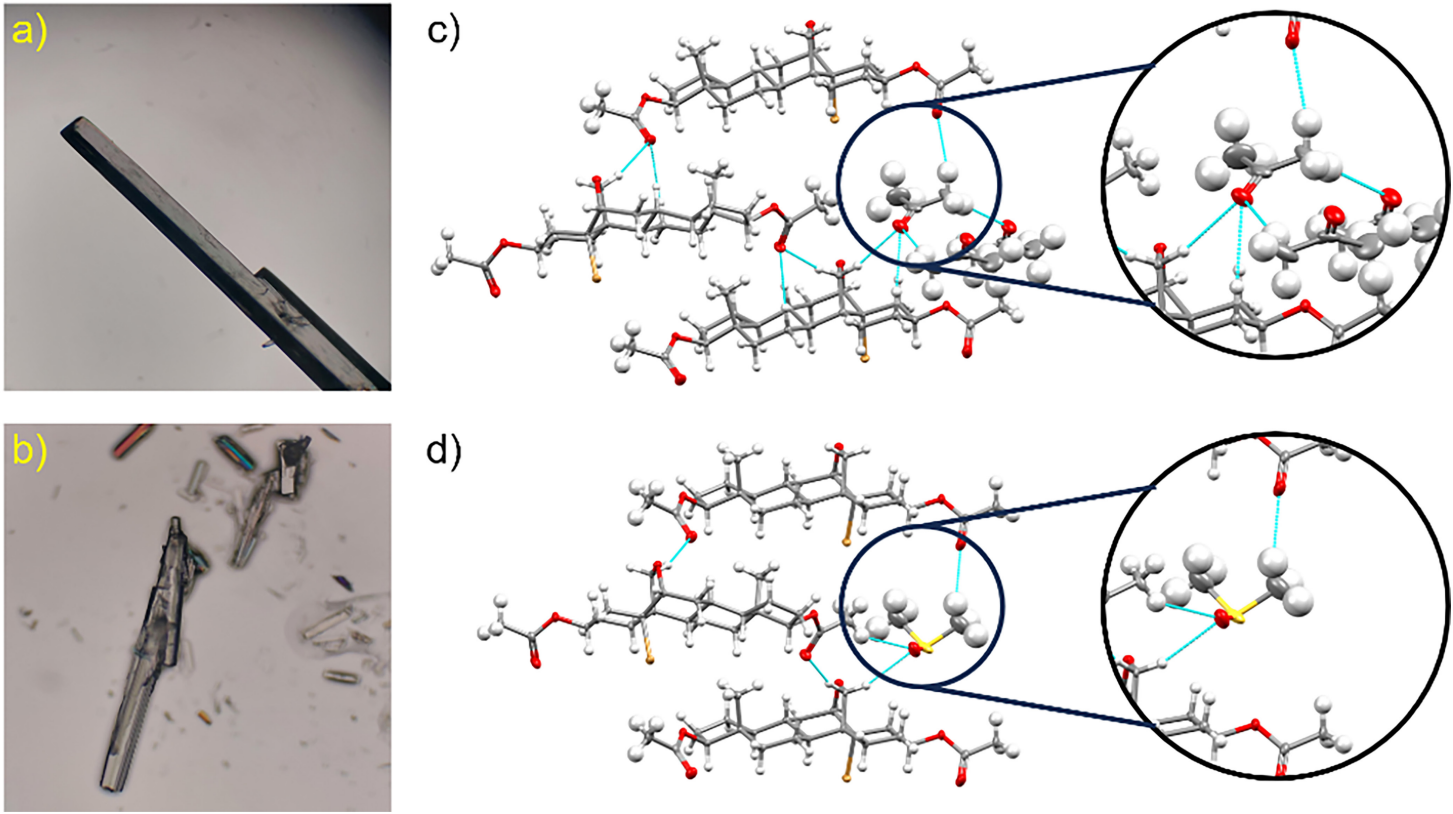
Microscope images of single crystals grown from (a) acetone and (b) DMSO. Representative close contacts between solvent molecules and steroidal fragments of **1** (c) Form **II** and (d) Form **III**, with only one of the two disordered DMSO molecules presented. Ellipsoids were drawn at 50% probability level.

As we continued our efforts to yield new crystalline forms, Compound **1** was also crystallized as solvates from saturated solutions of acetone (Form **II**) and DMSO (Form **III**) as prismatic‐shaped, transparent crystals, as depicted in Figure 3a,b. The SC‐XRD data allowed us to solve these structures in the orthorhombic *P*2_1_2_1_2_1_ space group. The cell parameters and calculated PXRD patterns (Figure S21) revealed isostructural crystals for both solvates (Tables S2–S3). The molecules of **1** in the crystalline structures are assembled by van der Waals interactions between the steroidal frameworks, through aliphatic C‐H and C‐H···O interactions between neighboring molecules. It is interesting to note that the bromine atom in both structures does not form halogen bonds with close molecules. Instead, the bromine atoms hold interactions with hydrogens in neighboring aliphatic chains. All these interactions are highlighted in Figure S24.

The solvent molecules in both Forms **II** and **III** are stabilized by the *sp* ^2^‐hybridized oxygens of ester groups towards the methyl groups of the acetone and DMSO molecules (Figure 3c,d). These moieties are placed in columnar arrays in the crystallographic a‐direction, and the molecules are aligned in opposite directions, as shown in Figure S25. During the crystallographic refinement of Form **III**, a SQUEEZE‐generated structure without the molecules of DMSO (Table S4) served as visual help to observe the channels described above, which are highlighted in Figure S26.

### 2.2 Phase Purity and Thermal Stability

**FIGURE 4:**
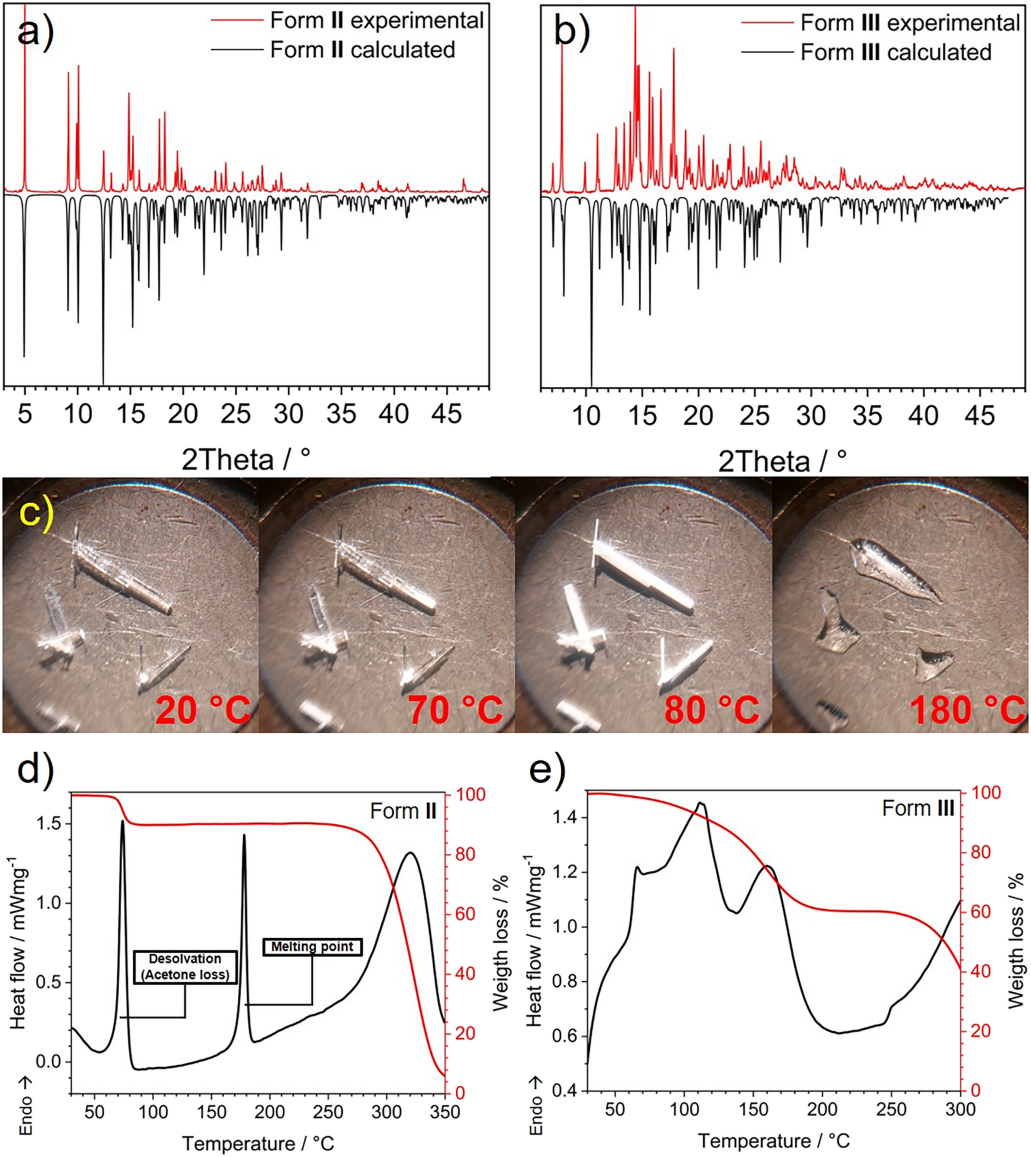
Powder X‐Ray diffractograms of (a) Form **II** and (b) Form **III**; (c) hot‐stage optical microscopy images of the desolvation process for Form **II** at several temperatures, starting from room temperature up to 180°C, finishing with the melting of the compound; Differential Scanning Calorimetry and Thermogravimetric Analysis (TGA/DSC) profiles of (d) Form **II** and (e) Form **III**.

The crystalline phase of these solids was confirmed through powder X‐ray diffraction (PXRD), and the experimental diffractograms of the three forms (Figures 4a,b and S22a) presented good agreement with the calculated patterns from single‐crystal X‐ray structures. Then, a series of progressive images were acquired to capture the physical changes in the single crystals of Form **II**. Figure 4c shows that the translucid, prismatic shapes at room temperature changed to opaque, white, prismatic shapes without observable structural collapse, in a direction from the tips towards the interior of the specimens. Furthermore, Differential Scanning Calorimetry and Thermogravimetric Analysis (DSC/TGA) experiments were also conducted. The resulting thermal profile of Form **II**, displayed in Figure 4d, reveals that for this solvate, the loss of acetone occurs at ~74°C with a mass loss of 10%. Afterward, the melting of the remaining solid of compound **1** was observed at 178°C, and a subsequent decomposition of the compound started at ~270°C, confirming the microscopy image observations.

We were curious to learn whether the channels formed by the solvent molecules could be maintained after the sample's thermal desolvation. For this, a sample of the acetone solvate was heated at 80°C for 30 min, and the material was submitted for PXRD characterization. The resulting diffractogram displayed in Figure S23 evidences that, instead of forming a porous crystal, the remaining powder migrated towards the solvent‐free Form **I** due to the heating treatment.

On the other hand, the thermal analysis for Form **III**, illustrated in Figure 4e, demonstrated that DMSO loses gradually but steadily from 60°C and onwards, concluding up to 180°C, a temperature remarkably close to the compound's melting point. Due to the high boiling point of the DMSO, which precludes its evaporation, we did not perform the microscopy images described for Form **II**.

**FIGURE 5:**
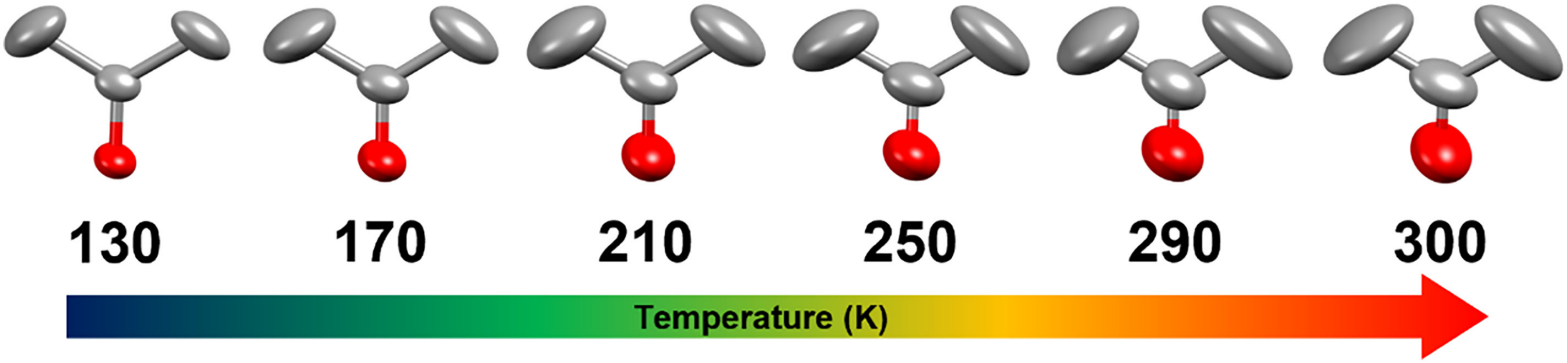
Elongation of the thermal ellipsoids in the molecule of acetone through the temperature interval (from 130 to 300 K) in Form **II**. The size of the ellipsoids from the methyl groups is more significant in comparison to the C=O group. Protons have been omitted for better visualization. The ellipsoids were drawn at 50% probability for each temperature.

The thermal behavior of Form **II** was also explored, looking for a hidden crystalline phase transition. For that matter, low‐temperature SC‐XRD experiments from 290 K down to 130 K, at 40 K intervals, were carried out (Tables S2 and S3). The analysis of the resulting data discarded the presence of any other phase transition. Interestingly, the shape and size of the thermal ellipsoids in the acetone molecules of this sample changed progressively as the temperature decreased, especially for the carbons of the methyl groups, in comparison to that of the carbonyl (C=O), which shows minor changes in that direction (Figure 5).

### 2.3 Dynamic Characterization of the Occluded Molecules

The observed changes through ^13^C CPMAS and SC‐XRD suggest that the molecules of solvent inside Forms **II** and **III** could be experiencing molecular motions inside the crystalline lattice, owing to its arrangements in the channels along the a‐crystallographic direction, as described above. This observation was derived from the shape similarities between acetone and DMSO. Microcrystalline samples of Forms **II** and **III** using acetone‐*d*_6_ and DMSO‐*d*_6_ were obtained to study the dynamics of the solvent molecules, respectively (samples were named Forms **II**‐*d*_*6*_ and **III**‐*d*_*6*_).

Afterward, we decided to employ ssNMR spectroscopy through the ^2^H quadrupolar spin‐echo pulse sequence at variable temperatures. Previous reports have established that due to the anisotropic distribution of charges in the deuterium nucleus derived from its quadrupolar nature (*I* = 1), the presence of molecular motions and different frequencies will provide a spectrum with distinctive line shapes within frequency intervals of 10^4^ and 10^7^ Hz (10 kHz to 10 MHz) [27].

**FIGURE 6:**
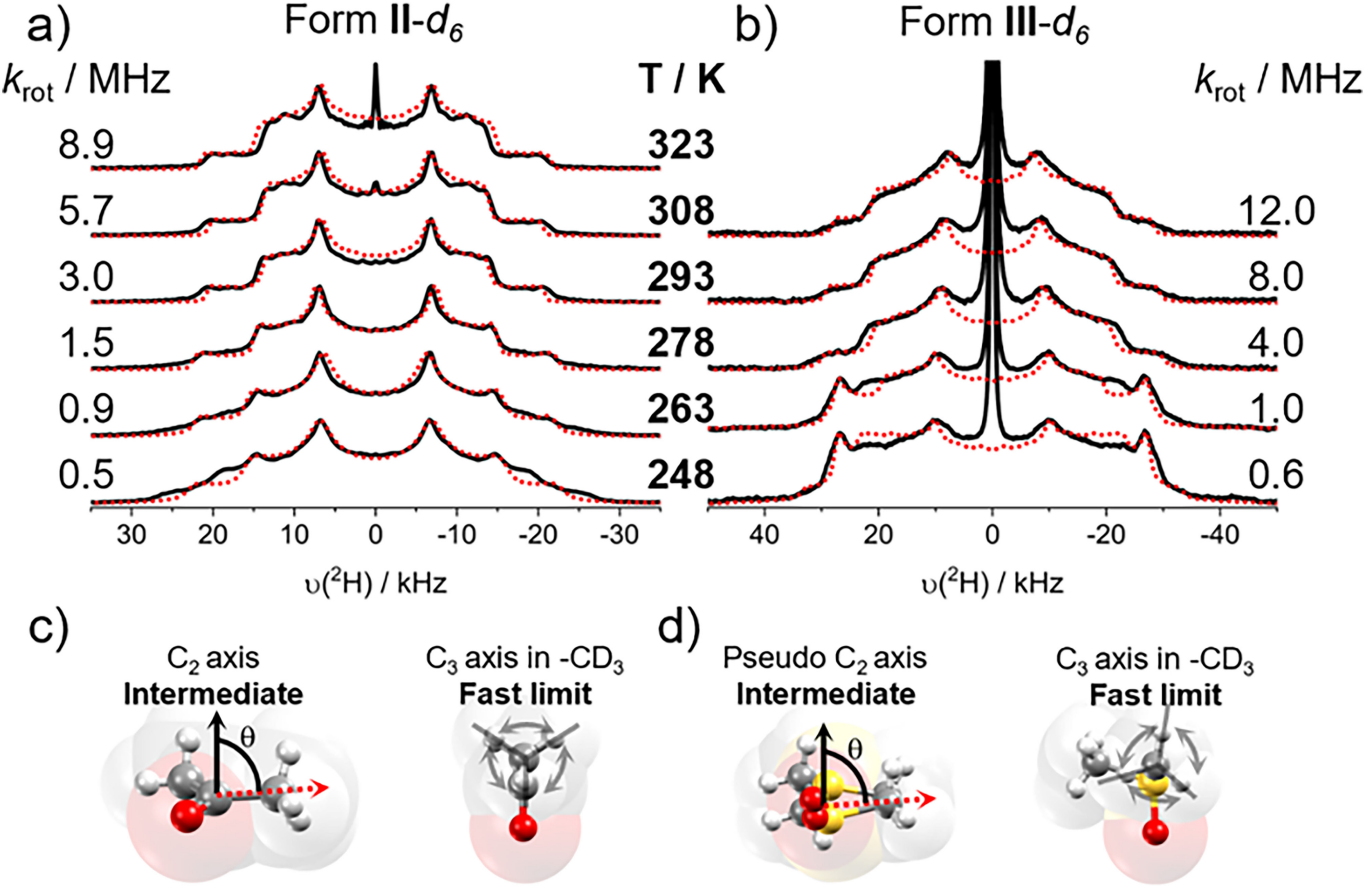
(a, b) Rotational dynamics of solvents in Forms **II**‐*d*_*6*_ and **III**‐*d*_*6*_ in variable‐temperature solid‐state quadrupolar spin‐echo ^2^H with experimental (solid black lines) and simulated (red dotted lines) spectra of acetone‐*d*_*6*_ and DMSO‐*d*_*6*_ with the associated rotational constant (*k*_rot_) for each temperature. (c, d) Principal motion modes for acetone‐*d*_*6*_ and DMSO‐*d*_*6*_ that generated the fitted deuterium line shapes.

The resulting deuterium spectra were obtained from 248 K (−25°C) to 323 K (50°C), as depicted in Figure 6a,b. For **II**‐*d*_*6*_, the change in the line shape corresponds to a narrowing of the spectrum, followed by the emergence of a sharp signal at 308 K and higher temperatures. This signal corresponds to the isotropic peak associated with the motion of the acetone molecules in random directions, which could occur due to the heating process that may influence the expelling of solvent molecules. To corroborate this hypothesis, a further experiment carried out at 338 K demonstrated the presence of a highly prominent isotropic peak at a temperature beyond that of the start of the desolvation process, which is visualized in the spectra in Figure S27.

For **III**‐*d*_*6*_, a prominent isotropic peak was observed even at temperatures as low as 248 K. This was attributed to the release of DMSO molecules on the surface of the microcrystalline powder.

The simulation of the spectra for both solvates was performed using NMR Weblab [28]. These simulations provide a picture of the dynamic processes related to experimental data and the rotational frequencies associated with motion. The complete data for the simulation of the spectra can be found in Table S5. The spectra of **II**‐*d*_*6*_ were simulated using quadrupolar coupling constants (QCC) for the C‐D bond of a methyl group between 54 and 64 kHz.

The rotational dynamics of the acetone molecules were decomposed into two components (Figure 6c): one associated with the rotation of the methyl groups around a C_3_‐axis (120° jump) within the fast limit, with a cone angle of θ = 74°. The second component corresponds to the flipping of the acetone along the C=O bond in a C_2_‐axis (180° jump) and cone angle of 56°. For both cases, the asymmetry parameter (*η*_Q_), a coefficient typically associated with the deviation of the motion of a molecule from a proposed axis [29], was set to nonzero values ranging from 0.75 to 0.5 as the experiment's temperature increased. This fact could potentially be associated with the presence of other types of movement (for instance, rocking) of the acetone molecules, which were not considered in the two‐component initial model. Nonetheless, the simulation of the deuterium spectra shows an agreement with the experimental line shape.

On the other hand, the simulation of the **III**‐*d*_*6*_ spectra was carried out using a QCC of 165 kHz for the C‐D coupling in DMSO‐*d*_*6*_, *η*_Q_ = 0, and the two‐component model, described in Figure 6d. The fast‐limit regime rotation of methyl groups was modeled with a C_3_‐axis, similar to that observed in Form **II**‐*d*_*6*_, using a cone angle θ = 70°. In contrast, the flipping motion of DMSO was modeled as a pseudo‐C_2_ movement, which is formally described as a fourfold movement with the distribution of equilibrium positions as 0.45/0.05/0.45/0.05, leading to the two most favored positions with a cone angle of 61°. Notably, the jump rate was modeled as a logarithmic Gaussian distribution of rotational frequencies at low temperatures. Despite the bent geometry of DMSO compared to acetone, the modeling of the rotational dynamics was similarly performed because the DMSO molecules show disorder over two sites at 180°, canceling the vectorial contributions of the bending and leading to an average position, as found in the acetone solvate.

**FIGURE 7:**
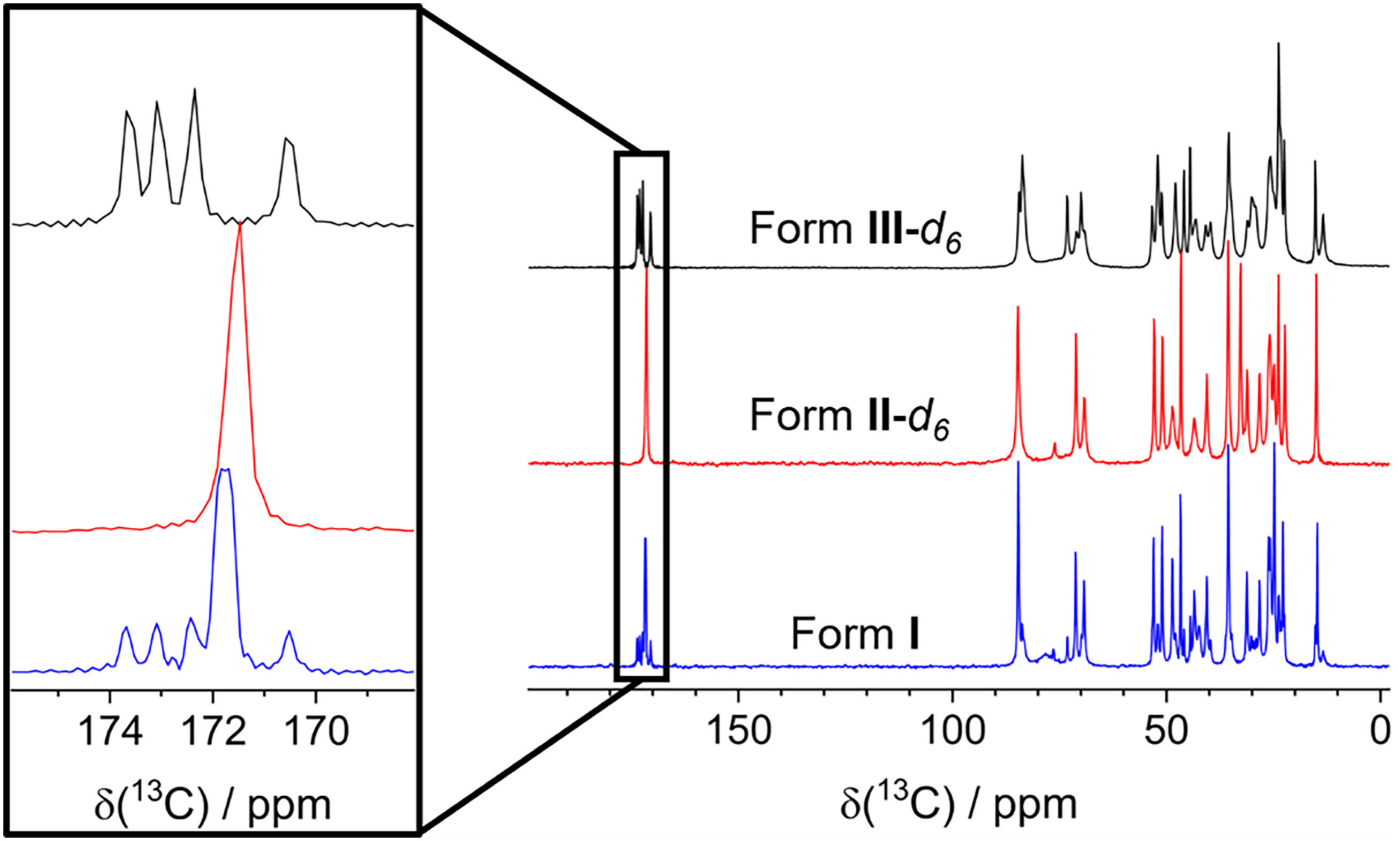
Comparison of the ^13^C CPMAS spectra of the three crystalline forms reported in this work, with the region between 176 and 168 ppm expanded for better visualization of the carbonyl signals.

Complementary characterization of microcrystalline samples of obtained Forms **I**, **II**‐*d*_*6*_, and **III**‐*d*_6_ was carried out via solid‐state Nuclear Magnetic Resonance (ssNMR), using the ^**1**3^C Cross‐Polarization Magic‐Angle Spinning (CP‐MAS) pulse sequence, which provided more information on the structural features of the samples. The carbonyl region in the ^13^C NMR spectrum of the solvent‐free form is consistent with the fact that two complete molecules of Compound **1** are present in the asymmetric unit, thus revealing up to four carbon signals in the 174–170 ppm region corresponding to the C=O of the acetates. Conversely, a significant overlapping of the carbonyl signals is observed in the spectrum of Form **II‐** *d*_*6*_ by the effect of the deuterated acetone, which is translated into a dominant single signal at ~172 ppm. It is important to note that the signal corresponding to the carbonyl fragment of the acetone‐*d*_*6*_ is not detected due to the lack of cross‐polarization ^1^H ➔ ^13^C. Moreover, the fast dynamics of acetone could compete with the relaxation times, further broadening and hiding the carbonyl signal [30]. These features are highlighted in the comparative insert in Figure 7.

### 2.4 Activation Energies, Enthalpic, and Entropic Factors Associated With Dynamics

The distribution of rotational frequencies for both solvent molecules resulted in similar values at low temperatures, ranging from 0.5 and 0.6 MHz for **II**‐*d*_*6*_ and **III**‐*d*_*6*_, respectively. In contrast, the high temperature regime displayed a higher interval of rotational frequencies for DMSO (~12.0 MHz at 308 K) and acetone (~8.9 MHz at 323 K). The determination of the rotational frequencies for each form through the temperature interval allowed the construction of their respective Arrhenius plots using the linear form of this equation (Equation 1).

(1) $$\ln\left(k_{\mathrm{rot}}\right)=-\frac{E_{a}}{R}\left(\frac{1}{T}\right)+\ln\left(A\right).$$

**FIGURE 8:**
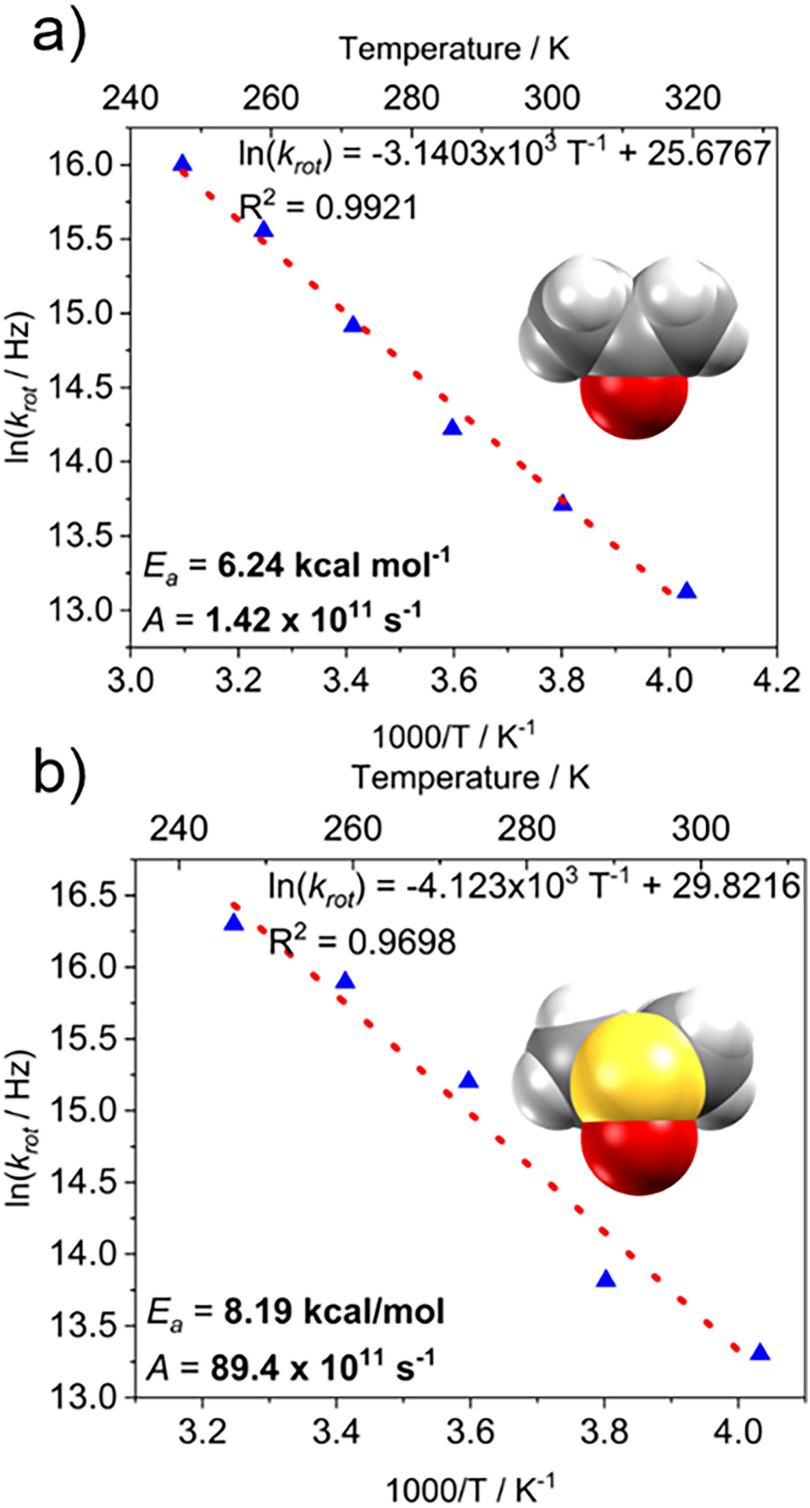
Arrhenius plots derived from rotational constants with the respective activation energy for rotation (*E*_a_) in each solvent.

This equation provided information on the energy of activation for rotation (*E*_a_) and the preexponential factor (*A*), both summarized in Table 1. From the data for **II**‐*d*_*6*_, it was determined that its rotational barrier is *E*_a_ = 6.24 kcal mol^−1^ with a preexponential factor of *A* = 1.42 × 10^11^ s^−1^ (Figure 8a), whereas for Form **III**‐*d*_*6*_ (Figure 8b), these parameters were determined as *E*_a_ = 8.19 kcal mol^−1^ and *A* = 89.4 × 10^11^ s^−1^. These values are very contrasting against each other because the preexponential factor found in Form **III** is 1 order of magnitude higher compared to that of Form **II**, which indicates that the motion of DMSO molecules is facilitated at low temperatures.

**TABLE 1:** Representative determined parameters from Arrhenius and Eyring models for the rotational dynamics of molecules inside Forms **II**‐*d*_*6*_ and **III**‐*d*_*6*_.

	Form **II**‐*d*_*6*_	Form **III**‐*d*_*6*_
*A* (×10^11^) (s^−1^)	1.42	89.4
*E*_a_ (kcal mol^−1^)	6.24	8.19
ΔH^≠^ (kcal mol^−1^)	5.7	7.6
ΔS^≠^ (cal mol^−1^ K^−1^)	−9.4	−1.1
Packing coefficient	0.684	0.715

Even more interestingly, the rotational barrier of Form **III** is higher than that of Form **II**. This phenomenon can be attributed to the higher packing coefficient found in the DMSO solvate **III**, which took a value of 0.715 at 130 K, whereas for the same temperature, the acetone solvate **II** was 0.684. A lighter crystal packing could result in a lower steric hindrance of the acetone molecules with its surrounding steroidal molecules, as can be confirmed by the number of noncovalent interactions compared to the DMSO in Form **III**.

To provide further insights into the transition state between equilibrium positions, the Transition State theory through the linear form of the Eyring equation was employed (Equation 2).

(2) $$\ln\left(\frac{k_{\mathrm{rot}}}{T}\right)=-\frac{∆H^{\neq }}{R}\left(\frac{1}{T}\right)+\frac{∆S^{\neq }}{R}+\ln\left(\frac{k_{B}}{h}\right).$$

By plotting the natural logarithm of *k*_rot_/T as a function of the reciprocal of the temperature, the slope and intercept provided information on the entropy (ΔH^≠^) and enthalpy (ΔS^≠^) of activation, respectively. The resulting Eyring plots are depicted in Figure S28, and the representative activation parameters are summarized in Table 1. The activation enthalpy values show minor variations between them; however, the activation entropy values were small and negative, suggesting that for both cases, the transition state from one‐to‐other equilibrium positions features an ordered transition. This may indicate some degree of correlation of this motion with the lattice vibrations inside these crystalline structures [31]. For **II**‐*d*_*6*_, with an ΔS^≠^ = −9.4 cal mol^−1^ K^−1^, the transition state requires a more ordered arrangement of molecules and noncovalent interactions, in comparison with the analog **III**‐*d*_*6*_, whose flipping was modeled as a pseudo *C*_2_ axis.

An additional description of these findings was provided by the calculation of interaction energies between pairs of molecules in both solvates using the *CrystalExplorer* software and the X‐ray crystal structures data collected at 130 K. The details of these calculations can be found in Section 4, and the features of the total energies are illustrated in Figure S29. In Form **II**, the lowest values for interaction energies are found between the acetone molecules and steroidal fragments, with −20.2 kJ mol^−1^, whereas the interaction between the solvent molecules (acetone‐acetone) is approximately −12.6 kJ mol^−1^. In sharp contrast, in Form **III**, the energy values between DMSO‐DMSO molecules are lower (approximately −33 kJ mol^−1^) than those between DMSO and the steroidal fragments (−11.5 kJ mol^−1^), indicating a stronger interaction between molecules of solvent than those found in Form **II**.

## 3 Conclusions

We have demonstrated that Compound **1** is prone to crystallize as a solvent‐free structure; however, crystallizations from acetone or DMSO yield isostructural solvate forms, which could be attributed to the similarity between the chemical structures of these solvents. Both portray a *sp* ^2^ oxygen, establishing noncovalent interactions (NCI) with hydrogens of the steroid skeleton.

The obtaining of the solvate Forms **II‐** *d*_*6*_ and **III‐** *d*_*6*_ using deuterated acetone and DMSO, respectively, allowed the study of the rotational dynamics of the solvent molecules within the crystalline packings. The results of this study revealed that the activation energy for rotation (*E*_a_) of the acetone in Form **II‐** *d*_*6*_ is lower than that for DMSO in Form **III‐** *d*_*6*_. Nonetheless, the rotational dynamics of DMSO molecules are facilitated by a combination of entropic factors, with additional rotation modes (pseudo‐*C*_2_ axis). This fact is visualized from the remarkably different preexponential factors (A) between crystalline forms, with that of Form **III** being over 60‐fold higher than that of Form **II**.

Moreover, we proposed that the presence of a higher quantity of NCI around the DMSO molecules and tighter crystalline packing are factors responsible for the higher activation barrier. Meanwhile, the acetone molecules in Form **II** require a collective movement of the crystal lattice to achieve the transition state required for the flipping movement, as deduced from the thermodynamic parameters obtained from the variable‐temperature ^2^H spectra.

## 4 Experimental Section

### 4.1 General Methods

Reactions were monitored by TLC on Alugram® SIL G/UV254 plates from Macherey‐Nagel. Chromatographic plates were sprayed with a 1% solution of vanillin in 50% HClO_4_ and heated until color developed. Melting points were measured on a Melt‐Temp II apparatus. Mass spectra were registered in a Thermo‐Electron spectrometer model DFS (Double Focus Sector). NMR spectra were recorded in CDCl_3_ solutions in Varian INOVA 400 or JNM‐ECZ600R spectrometers using the solvent signals 7.26 ppm for ^1^H and 77.00 ppm for ^13^C as references. All NMR spectra were recorded using the standard pulse sequences and parameters recommended by the manufacturer and were processed employing MestreNova (see http://mestrelab.com/). HRMS (APCI) spectra were registered on a PERKIN ELMER Model: AxION‐2 TOF MS spectrometer. Optical microscopy images were acquired using an Olympus BX43 microscope in a 4X zoom lens with a Samsung SM‐A525M (F1.8, 0.0ev, 1/60, 24 mm and ISO from 80 to 2000). For the preparation of synthetic precursors of the title compound **1**, see the Supporting Information.

### 4.2 Synthesis of 5α‐Bromo‐6β,19‐Epoxy‐Androstan‐3β,17β‐Diol Diacetate (1)

Steroidal alcohol **5** (1.12 g, 2.62 mmol) was dissolved in pyridine (5.5 mL). Acetic anhydride (1.25 mL) and a few crystals of 4‐dimethylaminopyridine (DMAP) were added, and the resulting reaction mixture was stirred at room temperature overnight. The reaction mixture was poured into water (20 mL), extracted with ethyl acetate (3 × 15 mL). The combined organic layers were washed with CuSO_4_ solution (4 × 20 mL), water (50 mL), dried over Na_2_SO_4_, and evaporated. The residue was purified in a chromatographic column packed with silica gel (20 g) employing a hexane/ethyl acetate mixture (9/1) for elution to afford 1.15 g (93.5%, 2.45 mmol) of acetylated product **1.** White solid, mp 178.4°C–179.6°C (recrystallized from hexane). ^1^H NMR (600 MHz, CDCl_3_) δ (ppm): 5.18 (tt, *J* = 11.3, 4.7 Hz, 1H, H‐3), 4.61 (dd, *J* = 9.2, 7.7 Hz, 1H, H‐17), 4.05 (d, *J* = 4.3 Hz, 1H, H‐6), 3.92 (d, *J* = 8.5 Hz, 1H, H‐19 *pro‐R*), 3.72 (d, *J* = 8.4 Hz, 1H, H‐19 *pro‐S*), 2.32 (ddd, *J* = 13.9, 4.6, 2.1 Hz, 1H, H‐12 eq.), 2.25 (dd, *J* = 13.9, 11.4 Hz, 1H, H‐12 ax.), 2.02 (s, 3H, CH_3_ acetyl), 2.01 (s, 3H, CH_3_ acetyl), 0.81 (s, 3H, H‐18). ^13^C{^1^H} NMR (150.91, CDCl_3_) δ (ppm): 23.3 C‐1, 26.8 C‐2, 69.8 C‐3, 41.2 C‐4, 74.2 C‐5, 82.1 C‐6, 32.3 C‐7, 33.1 C‐8, 48.6 C‐9, 45.9 C‐10, 22.1 C‐11, 36.7 C‐12, 43.3 C‐13, 48.8 C‐14, 22.9 C‐15, 27.5 C‐16, 82.3 C‐17, 12.4 C‐18, 67.5 C‐19, 171.0 C=O acetyl, 170.2 C=O acetyl, 21.1 CH_3_ acetyl, 21.2 CH_3_ acetyl. HRMS (APCI) *m*/*z:* [M + H]^+^ calcd for C_23_H_34_BrO_5_ 469.1584; found 469.1596.

### 4.3 X‐Ray Crystallography

The natural abundance variant of title Compound **1** and its deuterated‐solvent Forms **II** and **III** were crystallized from saturated solutions at room temperature. The crystals were mounted on glass fiber, and crystallographic data were collected at variable temperature for Form **II** and at 130 K for Forms **I** and **III** with an Oxford Diffraction Gemini diffractometer (λ_CuKα_ = 1.54184 Å, monochromator:graphite) with an Atlas CCD area detector. CrysAlisPro and CrysAlis RED software packages [32] were used for data collection and integration. The double‐pass method of scanning was used to exclude any noise. The collected frames were integrated by using an orientation matrix determined from the narrow‐frame scans. Final cell constants were determined by global refinement; collected data were corrected for absorbance by using analytical numeric absorption correction using a multifaceted crystal model based on expressions upon the Laue symmetry with equivalent reflections [33]. Structure solutions and refinement were carried out with the SHELXS‐2014 [34] and SHELXL‐2014 [35] packages. Mercury 4.0 [36] software was used to prepare material for publication. Full‐matrix least‐squares refinement was carried out by minimizing (Fo^2^−Fc^2^)^2^. All nonhydrogen atoms were refined anisotropically. H atoms of the O‐H group were located in a difference map and refined isotropically with *U*_iso_(H) = 1.5 *U*_eq_. H atoms attached to C atoms were placed in geometrically idealized positions and refined as riding on their parent atoms, with C−H = 0.95–1.00 Å with *U*_iso_(H) = 1.2*U*_eq_(C) for aromatic, methylene, and methyne groups and *U*_iso_(H) = 1.5*U*_eq_(C) for methyl groups. The crystallographic data for the structures reported in this paper has been deposited with the Cambridge Crystallographic Data Centre as supplementary publication numbers CCDC 2405470‐2405478. Copies of the data can be obtained free of charge upon application to CCDC, 12 Union Road, Cambridge, CB2 1EZ, UK (fax: [+44]1223‐336‐033, e‐mail: deposit@ccdc.cam.ac.uk).

### 4.4 Thermal Analysis

DSC and TGA experiments were performed in a Netzsch STA 449 F3 Jupiter in a coupled/simultaneous mode. Samples were placed as powders in 5‐mm aluminum crucibles with a hole in the cap. The heating ramp employed was 10°C/min under a nitrogen atmosphere. Hot‐stage microscopy images were captured using a hot‐stage Linkam LTS420E with a ramp program of 100°C/min and an Olympus BX43 optical microscope with a 4X zoom lens.

### 4.5 Solid‐State NMR

Solid‐state ^13^C with Cross‐Polarization Magic‐Angle Spinning (CP MAS) NMR spectra were obtained in a Bruker Avance II with a Larmor frequency of 500 MHz, equipped with a PH MAS DVT 500S1 BL3.2 N‐P/F‐H probe and Zirconia MAS rotors with VESPEL caps. The spectra were acquired by averaging 1000 to 2500 transients, MAS rate 12 kHz, delay time of 12 s, pulse width of 4 μs, and contact time of 2.5 ms at 20°C. Chemical shifts were referenced to adamantane (δ = 37.7 ppm) [37]. Solid‐state ^2^H echo‐spin experiments were performed on a Bruker Avance II at 76.7 MHz (deuterium resonance frequency) with a 3.2‐mm probe and 90° pulse of 2.9 μs. A quadrupolar‐echo sequence with phase recycling was used to suppress the undesired artifacts. An echo delay of 50 μs was used after the refocusing delay of 47 μs, and the recycling delay between pulses was 3 s. Thirty‐two scans were acquired for all temperatures explored. All spectra in this work were obtained with no line broadening for data processing.

### 4.6 Theoretical Calculations

Hirshfeld surface calculations and interaction energies were calculated using *CrystalExplorer 17* [38], using the *ce*‐B3LYP [39] 6‐31G(d,p) functional integrated in the software. Isosurfaces are displayed at 0.002 a.u. The scale factors for electrostatic, dispersion, polarization, and repulsion are reported by the authors and are as follows: *k*_elec_ = 1.057, *k*_disp_ = 0.740, *k*_pol_ = 0.871, and *k*_rep_ = 0.618.

## Conflicts of Interest

The authors declare no conflicts of interest.

## Supporting information


**Figure S1.** 3β‐Acetoxy‐5α‐bromo‐6β‐hydroxy‐androstan‐17‐one (3).
**Figure S2.** 1H NMR spectrum (600 MHz, CDCl3/CD3OD) of compound 3.
**Figure S3.** Sections of the 1H NMR spectrum (600 MHz, CDCl3/CD3OD) of compound 3.
**Figure S4.** 13C NMR spectrum (150.91 MHz, CDCl3/CD3OD) of compound 3.
**Figure S5.** Sections of the 13C NMR spectrum (150.91 MHz, CDCl3/CD3OD) of compound 3.
**Figure S6.** 3β‐Acetoxy‐5α‐bromo‐6β,19‐epoxy‐androstan‐17‐one (4).
**Figure S7.** 1H NMR spectrum (600 MHz, CDCl3) of compound 4.
**Figure S8.** Sections of the 1H NMR spectrum (600 MHz, CDCl3) of compound 4.
**Figure S9.** 13C NMR spectrum (150.91 MHz, CDCl3) of compound 4.
**Figure S10.** Sections of the 13C NMR spectrum (150.91 MHz, CDCl3) of compound 4.
**Figure S11.** 5α‐Bromo‐6β,19‐epoxy‐androstan‐3β,17β‐diol 3‐monoacetate (5).
**Figure S12.** 1H NMR spectrum (600 MHz, CDCl3) of compound 5.
**Figure S13.** Sections of the 1H NMR spectrum (600 MHz, CDCl3) of compound 5.
**Figure S14.** 13C NMR spectrum (150.91 MHz, CDCl3) of compound 5.
**Figure S15.** Sections of the 13C NMR spectrum (150.91 MHz, CDCl3) of compound 5.
**Figure S16.** 5α‐Bromo‐6β,19‐epoxy‐androstan‐3β,17β‐diol diacetate (1).
**Figure S17.** 1H NMR spectrum (600 MHz, CDCl3) of compound 1.
**Figure S18.** Sections of the 1H NMR spectrum (600 MHz, CDCl3) of compound 1.
**Figure S19.** 13C NMR spectrum (150.91 MHz, CDCl3) of compound 1.
**Figure S20.** Sections of the 13C NMR spectrum (150.91 MHz, CDCl3) of compound 1.
**Figures S21‐S23.** Powder X‐Ray Diffraction and Thermal Analysis.
**Figures S24‐S26.** Single Crystal X‐Ray Diffraction.
**Figure S27.** Solid‐State 2H Nuclear Magnetic Resonance.
**Figures S28‐S29.** Theoretical Calculations.

## Data Availability

The full data associated with this article are available in the Supporting Information.
