# Supplementary material for: Solid‐State NMR‐Assisted Dynamic Characterization of two Isostructural Solvates of 5α‐Bromo‐6β,19‐Epoxy‐Androstan‐3β,17β‐Diol Diacetate
Source: Magn Reson Chem. 2025 Feb 18;63(5-6):360–9. doi: 10.1002/mrc.5517 (PMC12052923; doi:10.1002/mrc.5517)
Supplement: Supplementary file 1 — Figure S1. 3β‐Acetoxy‐5α‐bromo‐6β‐hydroxy‐androstan‐17‐one (3). Figure S2. 1H NMR spectrum (600 MHz, CDCl3/CD3OD) of compound 3. Figure S3. Sections of the 1H NMR spectrum (600 MHz, CDCl3/CD3OD) of compound 3. Figure S4. 13C NMR spectrum (150.91 MHz, CDCl3/CD3OD) of compound 3. Figure S5. Sections of the 13C NMR spectrum (150.91 MHz, CDCl3/CD3OD) of compound 3. Figure S6. 3β‐Acetoxy‐5α‐bromo‐6β,19‐epoxy‐androstan‐17‐one (4). Figure S7. 1H NMR spectrum (600 MHz, CDCl3) of compound 4. Figure S8. Sections of the 1H NMR spectrum (600 MHz, CDCl3) of compound 4. Figure S9. 13C NMR spectrum (150.91 MHz, CDCl3) of compound 4. Figure S10. Sections of the 13C NMR spectrum (150.91 MHz, CDCl3) of compound 4. Figure S11. 5α‐Bromo‐6β,19‐epoxy‐androstan‐3β,17β‐diol 3‐monoacetate (5). Figure S12. 1H NMR spectrum (600 MHz, CDCl3) of compound 5. Figure S13. Sections of the 1H NMR spectrum (600 MHz, CDCl3) of compound 5. Figure S14. 13C NMR spectrum (150.91 MHz, CDCl3) of compound 5. Figure S15. Sections of the 13C NMR spectrum (150.91 MHz, CDCl3) of compound 5. Figure S16. 5α‐Bromo‐6β,19‐epoxy‐androstan‐3β,17β‐diol diacetate (1). Figure S17. 1H NMR spectrum (600 MHz, CDCl3) of compound 1. Figure S18. Sections of the 1H NMR spectrum (600 MHz, CDCl3) of compound 1. Figure S19. 13C NMR spectrum (150.91 MHz, CDCl3) of compound 1. Figure S20. Sections of the 13C NMR spectrum (150.91 MHz, CDCl3) of compound 1. Figures S21‐S23. Powder X‐Ray Diffraction and Thermal Analysis. Figures S24‐S26. Single Crystal X‐Ray Diffraction. Figure S27. Solid‐State 2H Nuclear Magnetic Resonance. Figures S28‐S29. Theoretical Calculations. [file MRC-63-360-s001.docx]

***Supporting Information***

**Solid-state NMR-Assisted Dynamic Characterization of Two Isostructural Solvates of 5α-Bromo-6β,19-epoxy-androstan-3β,17β -diol Diacetate**

**Josué Vazquez-Chavez^1^ | Armando Navarro-Huerta^2^ | Marcos Flores-Álamo^1^ | Braulio Rodríguez-Molina^2^** **| Martín A. Iglesias-Arteaga^1^ |**

^1^ Facultad de Química, Universidad Nacional Autónoma de México, 04510, Coyoacan, Mexico City, Mexico **|** ^2^ Instituto de Química, Universidad Nacional Autónoma de México, 04510, Coyoacan, Mexico City, Mexico

Josué Vazquez-Chavez and Armando Navarro-Huerta contributed equally.

**Correspondence:** Braulio Rodríguez-Molina ([brodriguez@iquimica.unam.mx](mailto:brodriguez@iquimica.unam.mx)) | Martín A. Iglesias-Arteaga ([martin.iglesias@unam.mx](mailto:martin.iglesias@unam.mx))

**Contents**

|  | Page |
| --- | --- |
| Synthesis of compound **1** | S3 |
| **Figure S1.** 3β-Acetoxy-5α-bromo-6β-hydroxy-androstan-17-one (**3**) | S6 |
| **Figure S2.** ^1^H NMR spectrum (600 MHz, CDCl_3_/CD_3_OD) of compound **3** | S7 |
| **Figure S3.** Sections of the ^1^H NMR spectrum (600 MHz, CDCl_3_/CD_3_OD) of compound **3** | S8 |
| **Figure S4.** ^13^C NMR spectrum (150.91 MHz, CDCl_3_/CD_3_OD) of compound **3**. | S9 |
| **Figure S5.** Sections of the ^13^C NMR spectrum (150.91 MHz, CDCl_3_/CD_3_OD) of compound **3** | S10 |
| **Figure S6.** 3β-Acetoxy-5α-bromo-6β,19-epoxy-androstan-17-one (**4**) | S11 |
| **Figure S7.** ^1^H NMR spectrum (600 MHz, CDCl_3_) of compound **4** | S12 |
| **Figure S8.** Sections of the ^1^H NMR spectrum (600 MHz, CDCl_3_) of compound **4** | S13 |
| **Figure S9.** ^13^C NMR spectrum (150.91 MHz, CDCl_3_) of compound **4** | S14 |
| **Figure S10.** Sections of the ^13^C NMR spectrum (150.91 MHz, CDCl_3_) of compound **4** | S15 |
| **Figure S11.** 5α-Bromo-6β,19-epoxy-androstan-3β,17β-diol 3-monoacetate (**5**) | S16 |
| **Figure S12.** ^1^H NMR spectrum (600 MHz, CDCl_3_) of compound **5** | S17 |
| **Figure S13.** Sections of the ^1^H NMR spectrum (600 MHz, CDCl_3_) of compound **5** | S18 |
| **Figure S14.** ^13^C NMR spectrum (150.91 MHz, CDCl_3_) of compound **5** | S19 |
| **Figure S15.** Sections of the ^13^C NMR spectrum (150.91 MHz, CDCl_3_) of compound **5** | S20 |
| **Figure S16.** 5α-Bromo-6β,19-epoxy-androstan-3β,17β-diol diacetate (**1**) | S21 |
| **Figure S17.** ^1^H NMR spectrum (600 MHz, CDCl_3_) of compound **1** | S22 |
| **Figure S18.** Sections of the ^1^H NMR spectrum (600 MHz, CDCl_3_) of compound **1** | S23 |
| **Figure S19.** ^13^C NMR spectrum (150.91 MHz, CDCl_3_) of compound **1** | S24 |
| **Figure S20.** Sections of the ^13^C NMR spectrum (150.91 MHz, CDCl_3_) of compound **1** | S25 |
| **Figures S21-S23.** Powder X-Ray Diffraction and Thermal Analysis | S26 |
| **Figures S24-S26.** Single Crystal X-Ray Diffraction | S27,28 |
| **Figure S27.** Solid-State ^2^H Nuclear Magnetic Resonance | S33 |
| **Figures S28-S29.** Theoretical Calculations | S35 |

**Synthesis of compound 1**

3β-Acetoxy-5α-bromo-6β-hydroxy-androstan-17-one (**3**)

Perchloric acid (0.27 M, 0.64 mL) and water (0.4 ml) were added to a stirred solution of dehydroepiandrosterone acetate **2** (661 mg, 2 mmol) in dioxane (12 mL). After cooling in a cold-water bath, *N*-bromoacetamide (331 mg, 2.4 mmol) was added slowly and the mixture was stirred for 2 h at room temperature in the dark. The reaction was quenched by the addition of Na_2_S_2_O_3_ solution (10 mL) and the product extracted with dichloromethane (3x20 mL). The combined organic layers were dried over Na_2_SO_4_, filtered, and evaporated. The residue was purified by crystallization from hexane/acetone to afford 470 mg (55.0%, 1.09 mmol) of the bromohydrin **3** as colorless needles. mp 175 °C (dec.) (from hexane/acetone) lit. ^S^^[[1]](#endnote-1)^ 173–175 °C (dec.). ^1^H NMR (600 MHz, CDCl_3_/CD_3_OD) δ (ppm): 5.45 (tt, *J* = 11.1, 5.7 Hz, 1H, H-3), 4.20 (t, *J* = 3.0 Hz, 1H, H-6), 2.49 (dd, *J* = 13.5, 10.6 Hz, 1H, H-4 ax.), 2.02 (s, 3H, CH_3_ acetyl), 1.33 (s, 3H, H-19), 0.86 (d, *J* = 3.0 Hz, 3H, H-18). ^13^C{^1^H} NMR (150.91, CDCl_3_/CD_3_OD) δ (ppm): 34.9 C-1, 26.1 C-2, 72.3 C-3, 38.1 C-4, 87.2 C-5, 74.6 C-6, 33.2 C-7, 30.2 C-8, 47.5 C-9, 40.4 C-10, 20.4 C-11, 31.1 C-12, 47.8 C-13, 50.7 C-14, 21.4 C-15, 35.7 C-16, 222.0 C-17, 13.6 C-18, 17.5 C-19, 170.9 C=O acetyl, 21.0 CH_3_ acetyl.

3β-Acetoxy-5α-bromo-6β,19-epoxy-androstan-17-one (**4**)

(Diacetoxyiodo)benzene (DIB) (966 mg, 3 mmol) and iodine (304 mg, 1.2 mmol) were added to a solution of the bromohydrin **3** (851 mg, 2 mmol) in cyclohexane (53 mL) and the reaction mixture was refluxed for 2 h under irradiation with two 100 W tungsten lamps. The purple reaction mixture was cooled to room temperature and quenched by addition of saturated aqueous Na_2_SO_3_ (until discoloration) and water. After extraction with EtOAc (3x50 mL) the combine organic layers were washed with brine (100 mL), dried over Na_2_SO_4_, filtered and evaporated. The residue was purified in a chromatographic column packed with silica gel (25 g) employing a hexane/ethyl acetate mixture (85/15) to afford 439 mg (51.6%, 1.03 mmol) of the bromo ether **4.** White solid, mp 184.7-187.1 °C (from hexane/ethyl acetate). ^1^H NMR (600 MHz, CDCl_3_) δ (ppm): 5.17 (ddt, *J* = 11.4, 7.0, 4.6 Hz, 1H, H-3), 4.09 (d, *J* = 4.6 Hz, 1H, H-6), 3.95 (dd, *J* = 8.7, 1.4 Hz, 1H, H-19 *pro-R*), 3.72 (d, *J* = 8.6 Hz, 1H, H-19 *pro-S*), 2.42 (dd, *J* = 18.6, 8.0 Hz, 1H, H-16), 2.33 (ddd, *J* = 13.8, 4.6, 2.2 Hz, 1H, H-12 eq.), 2.25 (dd, *J* = 13.8, 11.4 Hz, 1H, H-12 ax.), 2.01 (s, 3H, CH_3_ acetyl), 1.32 (td, *J* = 13.0, 4.1 Hz, 1H, H-7 eq.), 1.16 (td, *J* = 12.8, 3.9 Hz, 1H, H-11 ax.), 0.89 (s, 3H, H-18). ^13^C{^1^H} NMR (150.91, CDCl_3_) δ (ppm): 23.2 C-1, 26.8 C-2, 69.7 C-3, 41.2 C-4, 74.1 C-5, 82.0 C-6, 31.3 C-7, 33.0 C-8, 48.7 C-9, 46.0 C-10, 21.8 C-11, 31.8 C-12, 48.2 C-13, 49.4 C-14, 21.3 C-15, 35.6 C-16, 219.9 C-17, 14.1 C-18, 67.5 C-19, 170.2 C=O acetyl, 21.2 CH_3_ acetyl. HRMS (APCI) *m*/*z:* [M+H]^+^ calcd for C_21_H_30_BrO_4_ 425.1322; found 425.1304

5α-Bromo-6β,19-epoxy-androstan-3β,17β-diol 3-monoacetate (**5**)

NaBH_4_ (533 mg, 14.1 mmol) was added in portions to a stirred solution of steroidal ketone **4** (3 g, 7.05 mmol) in a methanol/dioxane mixture (28 mL/7 mL) at room temperature. The reaction mixture was stirred for 3 h, the solvents were removed under reduced pressure and the produced crude was extracted with ethyl acetate (3x30 mL), dried over Na_2_SO_4_, and the solvent evaporated. The residue was purified in a chromatographic column packed with silica gel (50 g) employing a hexane/ethyl acetate mixture (75/25) to afford 2.75 g (91.3%, 6.43 mmol) of the steroidal alcohol **5.** White solid, mp 126.7-128.3 °C (from hexane/ethyl acetate). ^1^H NMR (600 MHz, CDCl_3_) δ (ppm): 5.22–5.15 (m, 1H, H-3), 4.05 (d, *J* = 4.3 Hz, 1H, H-6), 3.93 (d, *J* = 8.7 Hz, 1H, H-19 *pro-R*), 3.74 (d, *J* = 8.3 Hz, 1H, H-19 *pro-S*), 3.67 (t, *J* = 8.5 Hz, 1H, H-17), 2.32 (ddd, *J* = 13.9, 4.6, 2.1 Hz, 1H, H-12 eq.), 2.26 (dd, *J* = 14.0, 11.3 Hz, 1H, H-12 ax.), 2.02 (s, 3H, CH_3_ acetyl), 0.77 (s, 3H, -18). ^13^C{^1^H} NMR (150.91, CDCl_3_) δ (ppm): 23.3 C-1, 26.8 C-2, 69.9 C-3, 41.3 C-4, 74.4 C-5, 82.1 C-6, 32.4 C-7, 33.4 C-8, 48.8 C-9, 45.9 C-10, 22.2 C-11, 36.5 C-12, 43.6 C-13, 49.1 C-14, 22.8 C-15, 30.5 C-16, 81.5 C-17, 11.4 C-18, 67.5 C-19, 170.3 C=O acetyl, 21.3 CH_3_ acetyl. HRMS (APCI) *m*/*z:* [M+H]^+^ calcd for C_21_H_32_BrO_4_ 427.1479; found 427.1474

**Figure S1**. 3β-Acetoxy-5α-bromo-6β-hydroxy-androstan-17-one (**3**)

**Figure S2.** ^1^H NMR spectrum (600 MHz, CDCl_3_/CD_3_OD) of compound **3**.

**Figure S3.** Sections of the ^1^H NMR spectrum (600 MHz, CDCl_3_/CD_3_OD) of compound **3.**

**Figure S4.** ^13^C NMR spectrum (150.91 MHz, CDCl_3_/CD_3_OD) of compound **3**.

**Figure S5.** Sections of the ^13^C NMR spectrum (150.91 MHz, CDCl_3_/CD_3_OD) of compound **3**.

**Figure S6.** 3β-Acetoxy-5α-bromo-6β,19-epoxy-androstan-17-one (**4**)

**Figure S7.** ^1^H NMR spectrum (600 MHz, CDCl_3_) of compound **4**.

**Figure S8.** Sections of the ^1^H NMR spectrum (600 MHz, CDCl_3_) of compound **4**.

**Figure S9.** ^13^C NMR spectrum (150.91 MHz, CDCl_3_) of compound **4**.

**Figure S10.** Sections of the ^13^C NMR spectrum (150.91 MHz, CDCl_3_) of compound **4**.

**Figure S11.** 5α-Bromo-6β,19-epoxy-androstan-3β,17β-diol 3-monoacetate (**5**)

**Figure S12.** ^1^H NMR spectrum (600 MHz, CDCl_3_) of compound **5**.

**Figure S13.** Sections of the ^1^H NMR spectrum (600 MHz, CDCl_3_) of compound **5**.

**Figure S14.** ^13^C NMR spectrum (150.91 MHz, CDCl_3_) of compound **5**.

**Figure S15.** Sections of the ^13^C NMR spectrum (150.91 MHz, CDCl_3_) of compound **5**.

**Figure S16.** 5α-bromo-6β,19-epoxy-androstan-3β,17β-diol diacetate (**1**).

**Figure S17.** ^1^H NMR spectrum (600 MHz, CDCl_3_) of compound **1**.

**Figure S18.** Sections of the ^1^H NMR spectrum (600 MHz, CDCl_3_) of compound **1**.

**Figure S19.** ^13^C NMR spectrum (150.91 MHz, CDCl_3_) of compound **1**.

**Figure S20.** Sections of the ^13^C NMR spectrum (150.91 MHz, CDCl_3_) of compound **1**

**Powder X-Ray Diffraction and Thermal Analysis**


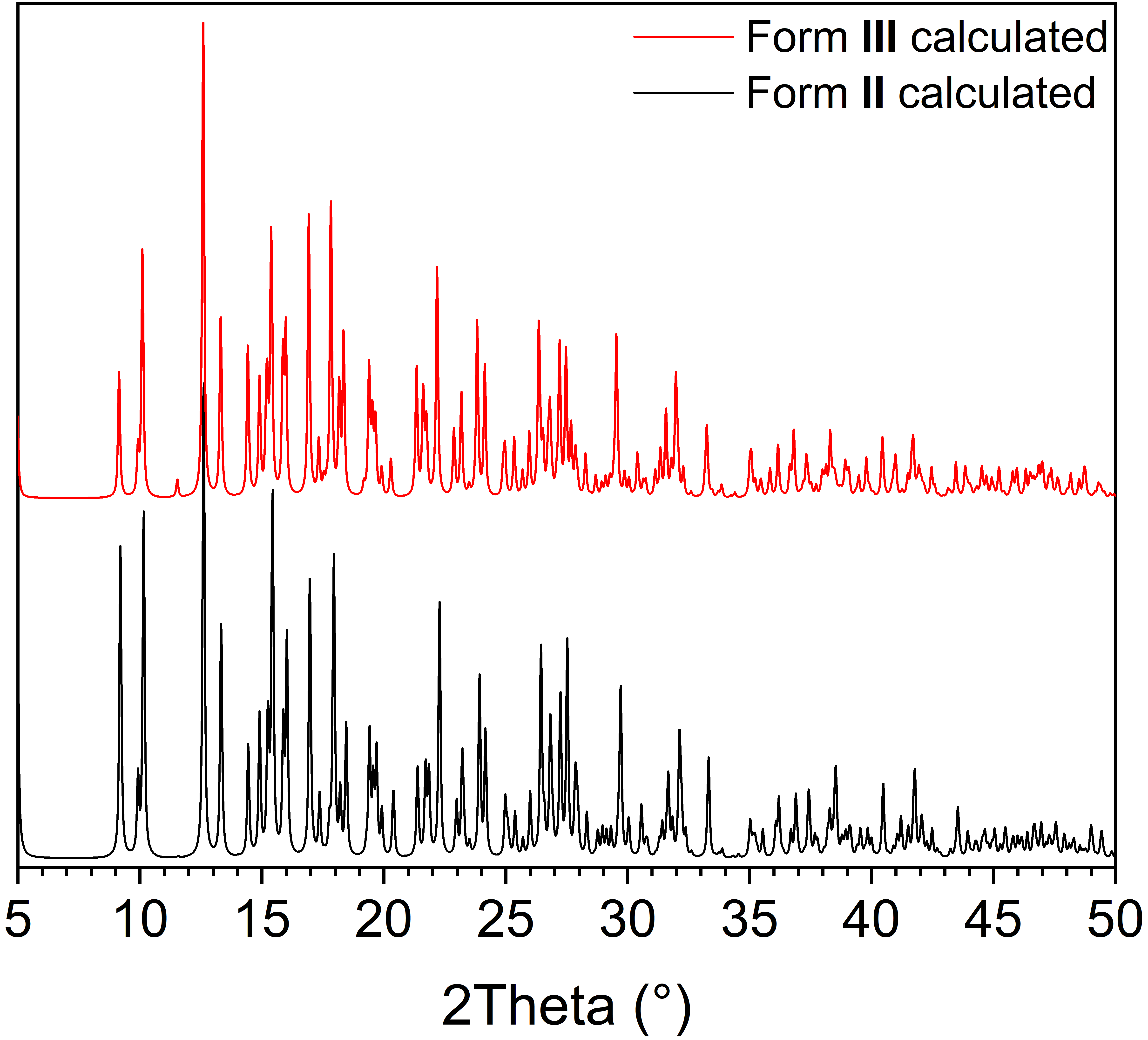


**Figure S21** Comparison of calculated PXRD from SC-XRD structures of Form **II** (acetone solvate) and Form **III** (DMSO solvate), highlighting the similarity between the crystalline forms.


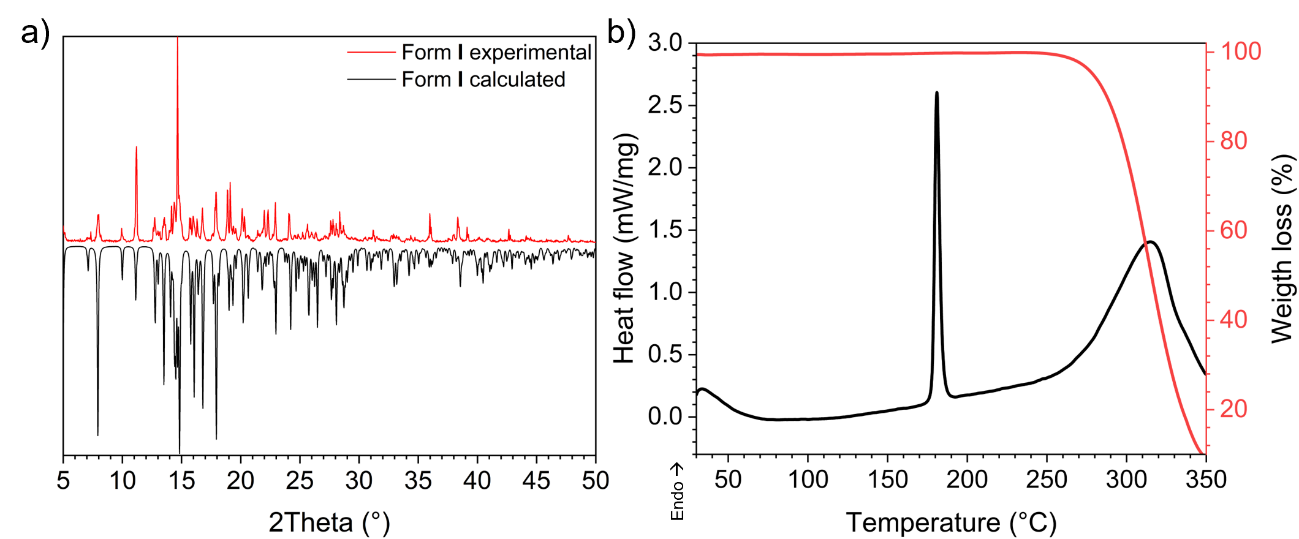


**Figure S22.** a) Comparative PXRD profile of Form **I** (experimental and calculated data from SC-XRD); b) DSC/TGA thermal profile of Form **I**.


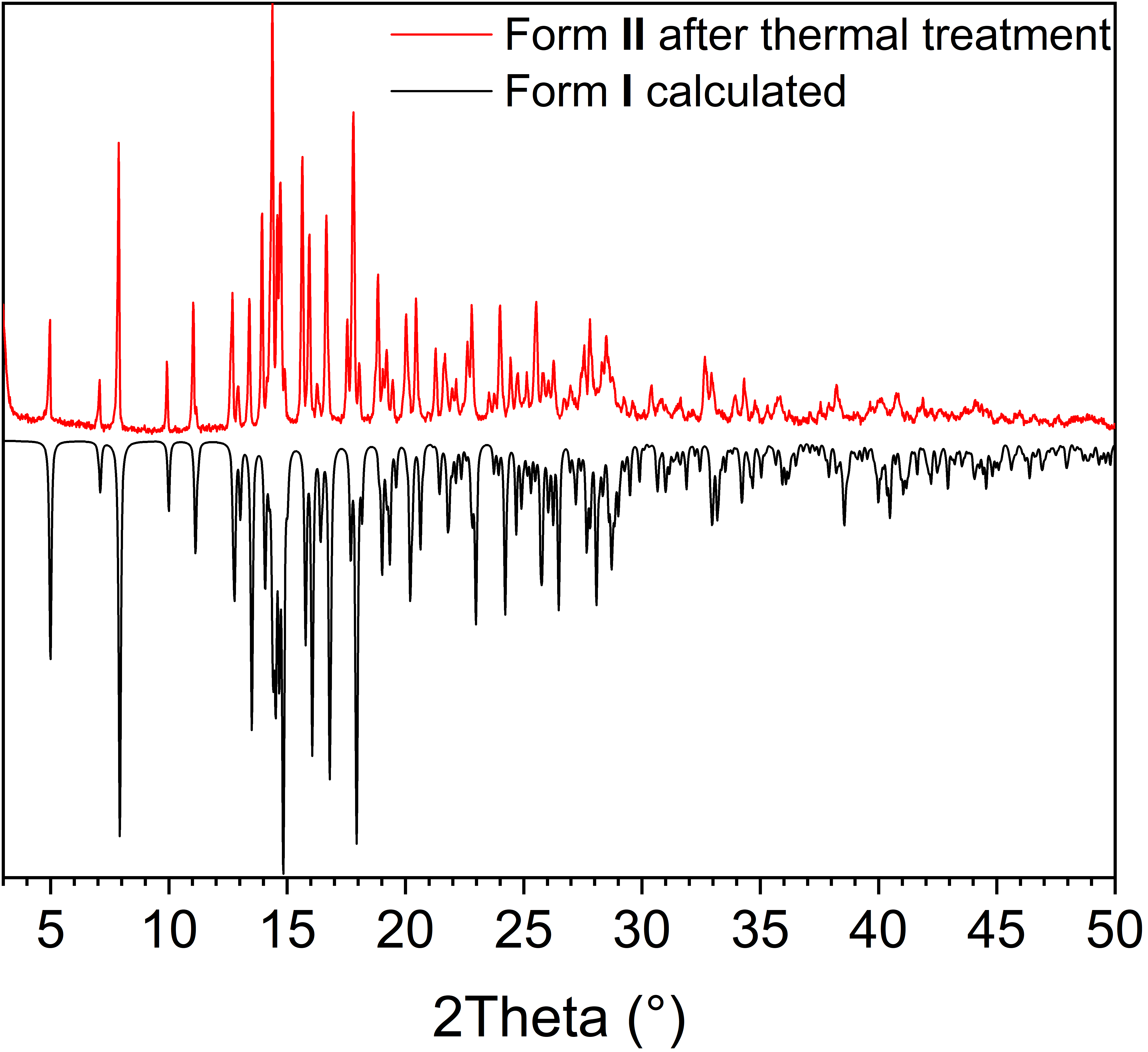


**Figure S23.** Comparative PXRD diffractogram of the microcrystalline powder of Form **II** after thermal treatment and subsequent solvent loss.

**Single Crystal X-Ray Diffraction**


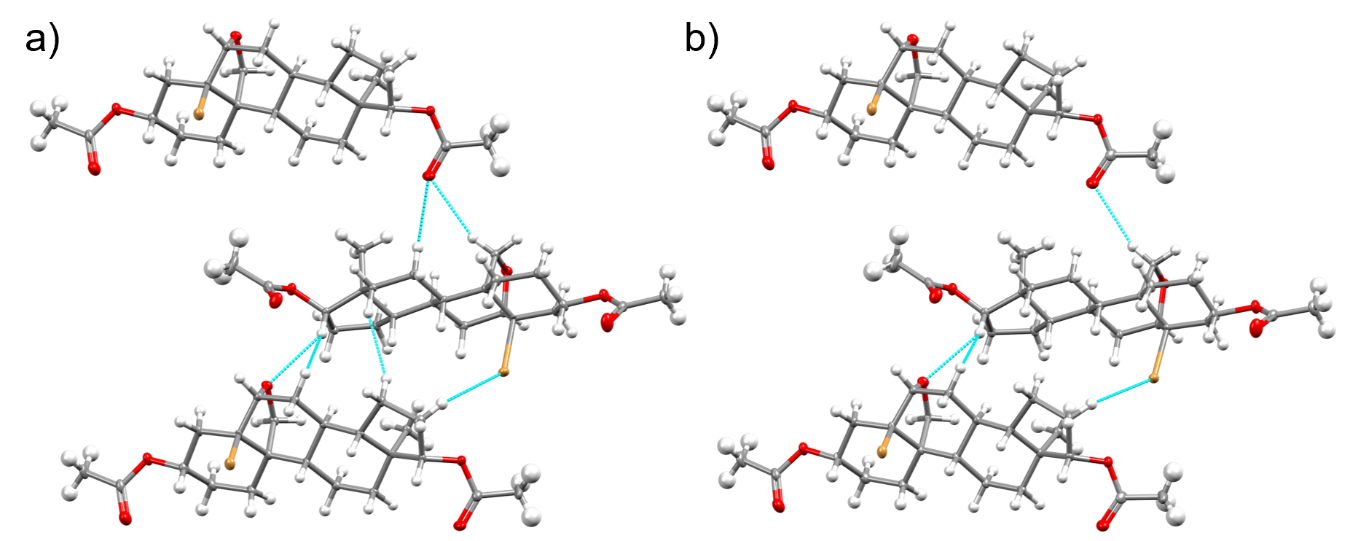


**Figure S24.** Representative close contacts between molecules of **1** towards the assembly of the crystalline network: a) Form **II**, b) Form **III**.


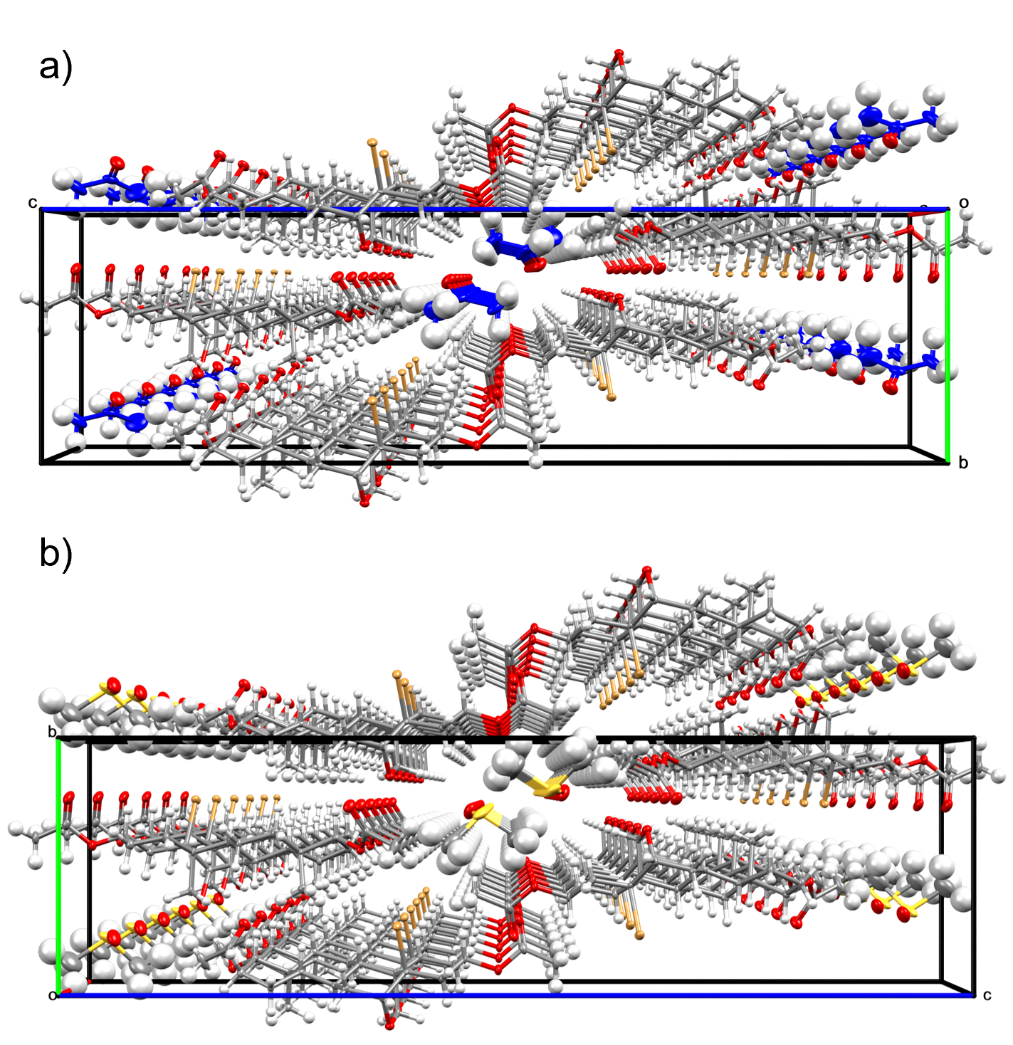


**Figure S25.** Crystalline arrangement showing the molecules of solvents through the crystal direction [100]: a) Form **II**, b) Form **III**.


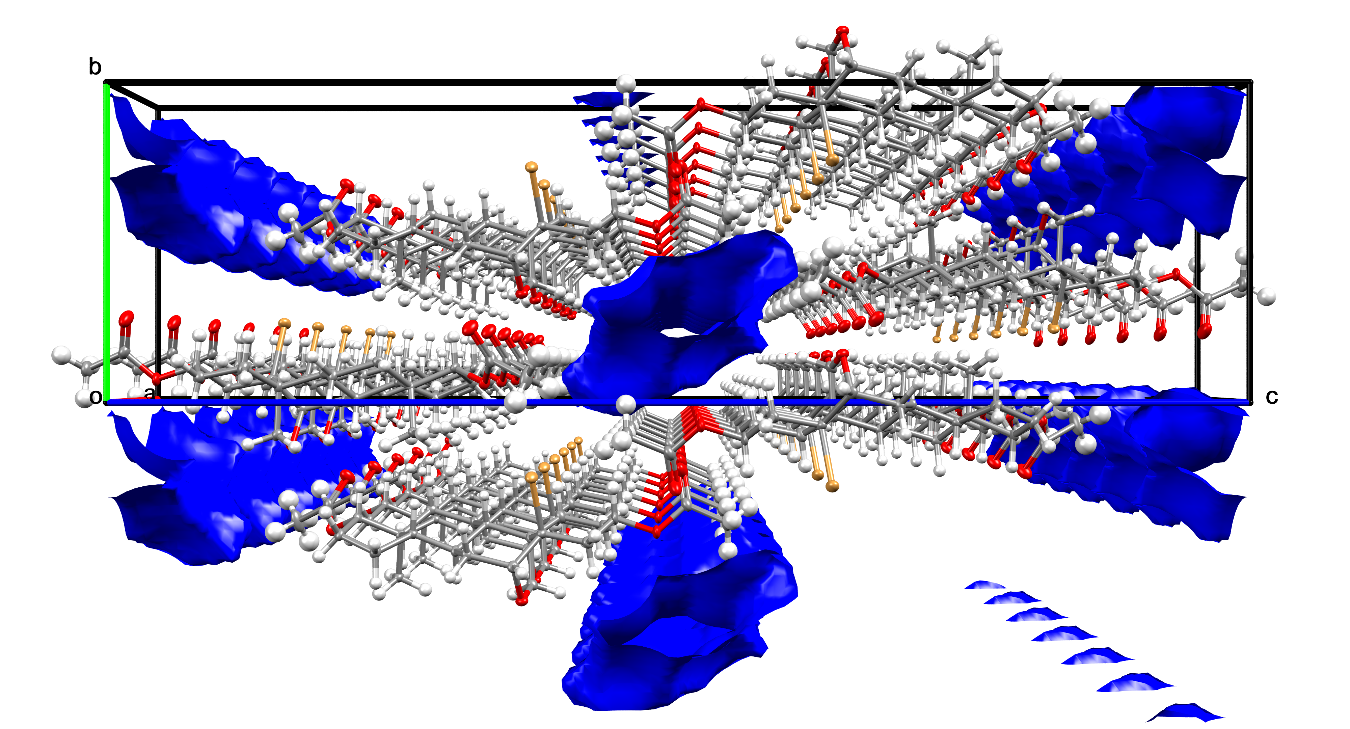


**Figure S26.** Generation of voids in a SQUEEZE structure of Form **III**, seen through the crystallographic direction [100]. The probe radius of the spere was 1.3 Å with a 0.3 Å grid spacing.

**Table S1.** Relevant crystallographic data

| Identification code | **Form I_Hexane** | |
| --- | --- | --- |
| Empirical formula | C_46_H_66_Br_2_O_10_ | |
| Formula weight | 938.80 | |
| Temperature [K] | 130(2) | |
| Wavelength [Å] | 0.71073 | |
| Crystal system | Orthorhombic | |
| Space group | *P*2_1_2_1_2_1_ | |
| Unit cell dimensions | a = 7.0598(4) Å | α= 90° |
|  | b = 24.9017(16) Å | β= 90° |
|  | c = 25.1617(18) Å | γ = 90° |
| Volume [Å^3^] | 4423.5(5) | |
| Z | 4 | |
| Density (calculated) [g cm^-3^] | 1.410 | |
| Absorption coefficient [mm^-1^] | 1.889 | |
| F(000) | 1968 | |
| Crystal size [mm] | 0.510 x 0.330 x 0.230 | |
| Theta range for data collection [°] | 3.629 to 29.522 | |
| Index ranges | -7 ≤ h ≤ 9  -33 ≤ k ≤ 31  -23 ≤ l ≤ 33 | |
| Reflections collected | 14743 | |
| Independent reflections | 9550 [R(int) = 0.0366] | |
| Completeness to theta = 25.242° | 99.5 % | |
| Refinement method | Full-matrix least-squares on F2 | |
| Data / restraints / parameters | 9550 / 0 / 529 | |
| Goodness-of-fit on F^2^ | 1.038 | |
| Final R indices [I>2sigma(I)] | R1 = 0.0461  wR2 = 0.0730 | |
| R indices (all data) | R1 = 0.0774  wR2 = 0.0848 | |
| Absolute structure parameter | -0.011(5) | |
| Extinction coefficient | n/a | |
| Largest diff. peak and hole [eÅ^-3^] | 0.410 and -0.545 | |
| CCDC deposition number | 2405470 | |

**Table S2.** Relevant crystallographic data

| Identification code | **Form-II-d6-130K** | | **Form-II-d6-170K** | | **Form-II-d6-210K** | |
| --- | --- | --- | --- | --- | --- | --- |
| Empirical formula | C_26_H_33_BrD_6_O_6_ | | C_26_H_33_BrD_6_O_6_ | | C_26_H_33_BrD_6_O_6_ | |
| Formula weight | 533.52 | | 533.52 | | 533.52 | |
| Temperature [K] | 130(2) | | 170(2) | | 210(2) | |
| Wavelength [Å] | 0.71073 | | 0.71073 | | 0.71073 | |
| Crystal system | Orthorhombic | | Orthorhombic | | Orthorhombic | |
| Space group | *P*2_1_2_1_2_1_ | | *P*2_1_2_1_2_1_ | | *P*2_1_2_1_2_1_ | |
| Unit cell dimensions | a = 7.1525(4) Å | α= 90° | a = 7.1814(4) Å | α= 90° | a = 7.2223(7) Å | α= 90° |
|  | b = 9.9746(4) Å | β= 90° | b = 10.0127(6) Å | β= 90° | b = 10.0474(9) Å | β= 90° |
|  | c = 35.6342(15) Å | γ = 90° | c = 35.695(3) Å | γ = 90° | c = 35.814(4) Å | γ = 90° |
| Volume [Å^3^] | 2542.2(2) | | 2566.7(3) | | 2598(4) | |
| Z | 4 | | 4 | | 4 | |
| Density (calculated) [g cm^-3^] | 1.394 | | 1.381 | | 1.364 | |
| Absorption coefficient [mm^-1^] | 1.655 | | 1.639 | | 1.619 | |
| F(000) | 1112 | | 1112 | | 1112 | |
| Crystal size [mm] | 0.530 x 0.270 x 0.140 | | 0.530 x 0.270 x 0.140 | | 0.530 x 0.270 x 0.140 | |
| Theta range for data collection [°] | 3.430 to 29.596 | | 3.425 to 29.593 | | 3.413 to 29.561 | |
| Index ranges | -9 ≤ h ≤ 9 -13 ≤ k ≤ 12 -49 ≤ l ≤ 47 | | -9 ≤ h ≤ 9 -9 ≤ k ≤ 12 -47 ≤ l ≤ 48 | | -9 ≤ h ≤ 6 -11 ≤ k ≤ 12 -48 ≤ l ≤ 48 | |
| Reflections collected | 16159 | | 13332 | | 16676 | |
| Independent reflections | 6104 [R(int) = 0.0377] | | 5892 [R(int) = 0.0439] | | 6159 [R(int) = 0.0421] | |
| Completeness to theta = 25.242° | 99.6% | | 99.7% | | 99.7% | |
| Refinement method | Full-matrix least-squares on F^2^ | | Full-matrix least-squares on F^2^ | | Full-matrix least-squares on F^2^ | |
| Data / restraints / parameters | 6104 / 0 / 301 | | 5892 / 0 / 301 | | 6159 / 0 / 301 | |
| Goodness-of-fit on F^2^ | 1.041 | | 1.105 | | 1.094 | |
| Final R indices [I>2sigma(I)] | R1 = 0.0372 wR2 = 0.0683 | | R1 = 0.0499 wR2 = 0.0814 | | R1 = 0.0470 wR2 = 0.0774 | |
| R indices (all data) | R1 = 0.0450 wR2 = 0.0730 | | R1 = 0.0643 wR2 = 0.0901 | | R1 = 0.0633 wR2 = 0.0828 | |
| Absolute structure parameter | 0.003(4) | | -0.004(6) | | -0.016(5) | |
| Extinction coefficient | n/a | | n/a | | n/a | |
| Largest diff. peak and hole [eÅ^-3^] | 0.295 and -0.629 | | 0.699 and -0.658 | | 0.399 and -0.597 | |
| CCDC deposition number | 2405471 | | 2405472 | | 2405473 | |

**Table S3.** Relevant crystallographic data

| Identification code | **Form-II-d6-250K** | | **Form-II-d6-290K** | | **Form-II-d6-300K** | |
| --- | --- | --- | --- | --- | --- | --- |
| Empirical formula | C_26_H_33_BrD_6_O_6_ | | C_26_H_33_BrD_6_O_6_ | | C_26_H_33_BrD_6_O_6_ | |
| Formula weight | 533.52 | | 533.52 | | 533.52 | |
| Temperature [K] | 250(2) | | 290(2) | | 290(2) | |
| Wavelength [Å] | 0.71073 | | 0.71073 | | 0.71073 | |
| Crystal system | Orthorhombic | | Orthorhombic | | Orthorhombic | |
| Space group | *P*2_1_2_1_2_1_ | | *P*2_1_2_1_2_1_ | | *P*2_1_2_1_2_1_ | |
| Unit cell dimensions | a = 7.205(3) Å | α= 90° | a = 7.2698(8) Å | α= 90° | a = 7.2796(8) Å | α= 90° |
|  | b = 10.096(3) Å | β= 90° | b = 10.1082(13) Å | β= 90° | b = 10.1029(18) Å | β= 90° |
|  | c = 35.99(3) Å | γ = 90° | c = 35.877(4) Å | γ = 90° | c = 35.696(4) Å | γ = 90° |
| Volume [Å^3^] | 2618.(3) | | 2636.4(5) | | 2625.2(6) | |
| Z | 4 | | 4 | | 4 | |
| Density (calculated) [g cm^-3^] | 1.353 | | 1.344 | | 1.350 | |
| Absorption coefficient [mm^-1^] | 1.607 | | 1.596 | | 1.603 | |
| F(000) | 1112 | | 1112 | | 1112 | |
| Crystal size [mm] | 0.530 x 0.270 x 0.140 | | 0.530 x 0.270 x 0.140 | | 0.590 x 0.380 x 0.280 | |
| Theta range for data collection [°] | 3.396 to 29.552 | | 3.498 to 29.626 | | 3.494 to 29.561 | |
| Index ranges | -8 ≤ h ≤ 9 -13 ≤ k ≤ 13 -37 ≤ l ≤ 47 | | -9 ≤ h ≤ 9 -13 ≤ k ≤ 13 -49 ≤ l ≤ 48 | | -9 ≤ h ≤ 8 -14 ≤ k ≤ 13 -47 ≤ l ≤ 49 | |
| Reflections collected | 16996 | | 16057 | | 17027 | |
| Independent reflections | 6188 [R(int) = 0.0408] | | 6146 [R(int) = 0.0764] | | 6324 [R(int) = 0.0558] | |
| Completeness to theta = 25.242° | 99.6% | | 99.6% | | 99.6% | |
| Refinement method | Full-matrix least-squares on F^2^ | | Full-matrix least-squares on F^2^ | | Full-matrix least-squares on F^2^ | |
| Data / restraints / parameters | 6188 / 0 / 301 | | 6146 / 0 / 301 | | 6324 / 0 / 301 | |
| Goodness-of-fit on F^2^ | 1.075 | | 0.987 | | 1.154 | |
| Final R indices [I>2sigma(I)] | R1 = 0.0442 wR2 = 0.0727 | | R1 = 0.0537 wR2 = 0.0866 | | R1 = 0.0644 wR2 = 0.1002 | |
| R indices (all data) | R1 = 0.0706 wR2 = 0.0814 | | R1 = 0.1039 wR2 = 0.1137 | | R1 = 0.1013 wR2 = 0.1150 | |
| Absolute structure parameter | 0.001(5) | | 0.002(10) | | 0.023(7) | |
| Extinction coefficient | n/a | | n/a | | n/a | |
| Largest diff. peak and hole [eÅ^-3^] | 0.350 and -0.463 | | 0.394 and -0.508 | | 0.503 and -0.814 | |
| CCDC deposition number | 2405474 | | 2405475 | | 2405476 | |

**Table S4.** Relevant crystallographic data

| Identification code | **Form III-n** | | **Form III-sq** | |
| --- | --- | --- | --- | --- |
| Empirical formula | C_25_H_39_BrO_6_S | | C_23_H_33_BrO_5_ | |
| Formula weight | 547.53 | | 469.40 | |
| Temperature [K] | 130(2) | | 130(2) | |
| Wavelength [Å] | 0.71073 | | 0.71073 | |
| Crystal system | Orthorhombic | | Orthorhombic | |
| Space group | *P*2_1_2_1_2_1_ | | *P*2_1_2_1_2_1_ | |
| Unit cell dimensions | a = 7.1629(7) Å | α= 90° | a = 7.1629(7) Å | α= 90° |
|  | b = 10.0383(11) Å | β= 90° | b = 10.0383(11) Å | β= 90° |
|  | c = 35.647(5) Å | γ = 90° | c = 35.647(5) Å | γ = 90° |
| Volume [Å^3^] | 2563.2(5) | | 2563.2(5) | |
| Z | 4 | | 4 | |
| Density (calculated) [g cm^-3^] | 1.419 | | 1.216 | |
| Absorption coefficient [mm^-1^] | 1.723 | | 1.630 | |
| F(000) | 1152 | | 984 | |
| Crystal size [mm] | 0.420 x 0.190 x 0.180 | | 0.420 x 0.190 x 0.180 | |
| Theta range for data collection [°] | 3.429 to 29.875 | | 3.429 to 29.875 | |
| Index ranges | -9 ≤ h ≤ 9  -13 ≤ k ≤ 13  -47 ≤ l ≤ 43 | | -9 ≤ h ≤ 9  -13 ≤ k ≤ 13  -47 ≤ l ≤ 43 | |
| Reflections collected | 16764 | | 16764 | |
| Independent reflections | 6162 [R(int) = 0.0548] | | 6162 [R(int) = 0.0548] | |
| Completeness to theta = 25.242° | 99.7 % | | 99.7 % | |
| Refinement method | Full-matrix least-squares on F2 | | Full-matrix least-squares on F2 | |
| Data / restraints / parameters | 6162 / 6 / 318 | | 6162 / 0 / 265 | |
| Goodness-of-fit on F^2^ | 1.033 | | 1.025 | |
| Final R indices [I>2sigma(I)] | R1 = 0.0483  wR2 = 0.0849 | | R1 = 0.0472  wR2 = 0.0894 | |
| R indices (all data) | R1 = 0.0696  wR2 = 0.0940 | | R1 = 0.0655  wR2 = 0.0972 | |
| Absolute structure parameter | -0.006(6) | | 0.007(6) | |
| Extinction coefficient | n/a | | n/a | |
| Largest diff. peak and hole [eÅ^-3^] | 0.749 and -0.725 | | 0.623 and -0.600 | |
| CCDC deposition number | 2405477 | | 2405478 | |

**Solid-State ^2^H Nuclear Magnetic Resonance**





**Figure S27.** ssNMR ^2^H quadrupolar echo-spin spectrum of **II**-d_6_ at 338 K, displaying an isotropic peak due to the loss of deuterated acetone above the desolvation temperature.

**Table S5.** Complete parameters for simulation of quadrupolar ^2^H spin-echo spectra of **II**-d_6_ and **III**-d_6_.

| Temperature (K) | Parameter | **II**-*d_6_* | | **III**-*d_6_* | |
| --- | --- | --- | --- | --- | --- |
|  |  | Modes of motion | | Modes of motion | |
| 248 | *I* | 1 | | 1 | |
|  | QCC (kHz) | 55 | | 165 | |
|  | *η* | 0.75 | | 0 | |
|  | Timescale | Intermediate | | Fast limit | Intermediate |
|  | Type | 2-fold | | 3-fold | 4-fold |
|  | θ (°) | 56 | | 70.5 | 62.5 |
|  | Populations | N/A | | N/A | 0.47/0.03/0.47/0.03 |
|  | *k*_rot_ (MHz) | 0.5 | | N/A | 0.6 |
| 263 | *I* | 1 | | 1 | |
|  | QCC (kHz) | 64 | | 165 | |
|  | *η* | 0.8 | | 0 | |
|  | Timescale | Intermediate | | Fast limit | Intermediate |
|  | Type | 2-fold | | 3-fold | 4-fold |
|  | θ (°) | 54 | | 70.5 | 62.5 |
|  | Populations | N/A | | N/A | 0.44/0.06/0.44/0.06 |
|  | *k*_rot_ (MHz) | 0.9 | | N/A | 1.0 |
| 278 | *I* | 1 | | 1 | |
|  | QCC (kHz) | 58 | | 165 | |
|  | *η* | 0.75 | | 0 | |
|  | Timescale | Intermediate | | Fast limit | Intermediate |
|  | Type | 2-fold | | 3-fold | 4-fold |
|  | θ (°) | 55 | | 70.5 | 61 |
|  | Populations | N/A | | N/A | 0.44/0.06/0.44/0.06 |
|  | *k*_rot_ (MHz) | 1.5 | | N/A | 4.0 |
| 293 | *I* | 1 | | 1 | |
|  | QCC (kHz) | 54 | | 165 | |
|  | *η* | 0.75 | | 0 | |
|  | Timescale | Intermediate | | Fast limit | Intermediate |
|  | Type | 2-fold | | 3-fold | 4-fold |
|  | θ (°) | 55 | | 70.5 | 61 |
|  | Populations | N/A | | N/A | 0.45/0.05/0.45/0.05 |
|  | *k*_rot_ (MHz) | 3.0 | | N/A | 8.0 |
| 308 | *I* | 1 | | 1 | |
|  | QCC (kHz) | 56 | | 165 | |
|  | *η* | 0.50 | | 0 | |
|  | Timescale | Intermediate | | Fast limit | Intermediate |
|  | Type | 2-fold | | 3-fold | 4-fold |
|  | θ (°) | 53 | | 70.5 | 60 |
|  | Populations | N/A | | N/A | 0.45/0.05/0.45/0.05 |
|  | *k*_rot_ (MHz) | 5.7 | | N/A | 12.0 |
| 323 | *I* | 1 | | N/A | |
|  | QCC (kHz) | 54 | |  |  |
|  | *η* | 0.35 | |  |  |
|  | Timescale | Fast limit | Intermediate |  |  |
|  | Type | 3-fold | 2-fold |  |  |
|  | θ (°) | 74 | 65 |  |  |
|  | Populations | N/A | N/A |  |  |
|  | *k*_rot_ (MHz) | N/A | 8.9 |  |  |

**Theoretical Calculations**


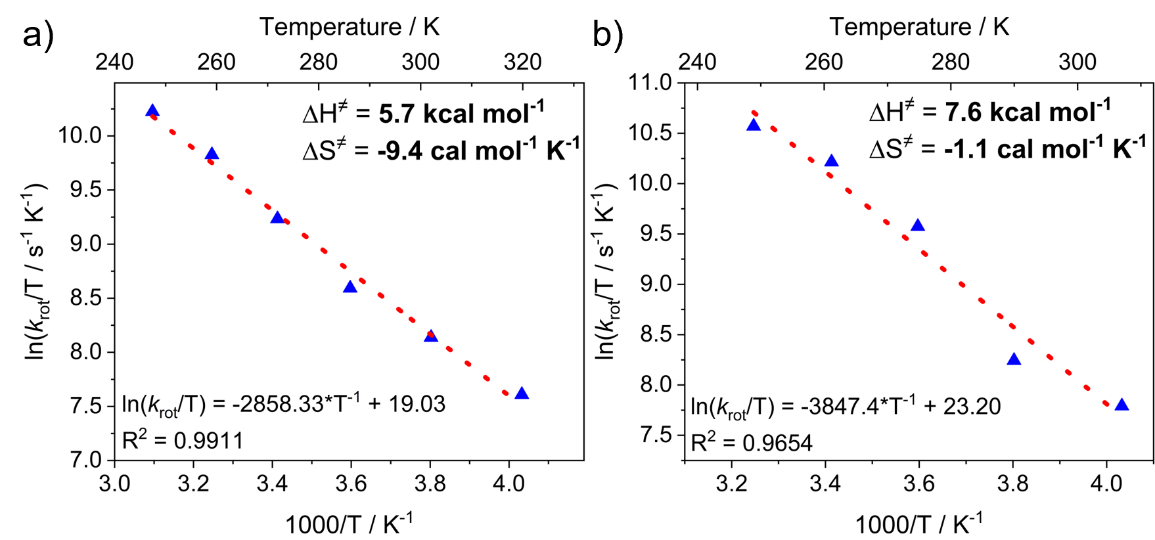


**Figure S28.** Eyring plots of a) **II**-d_6_ and b) **III**-d_6_ with their respective activation parameters as insets.


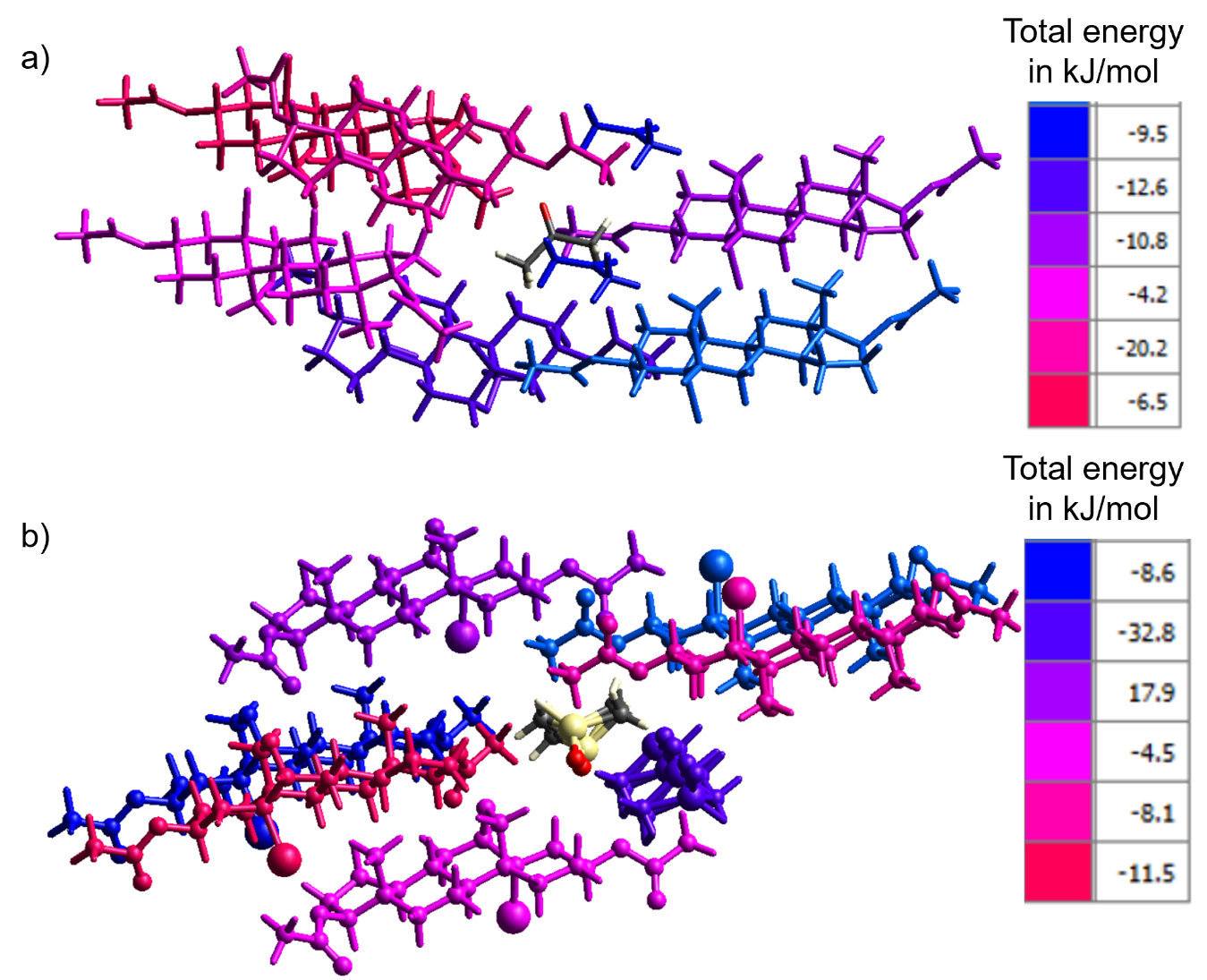


**Figure S29.** Depiction of total energy calculated around solvent molecules with neighboring molecules for a) **II**-d_6_ and b) **III**-d_6_ with their respective energy values and color codes.

1. ^S^ Grenville, V.; Patel, D.K.; Petrow, V.; Stuart-Webb, I.A.; Williamson, D.M. Modified Steroid Hormones. Part VI. Further 6-Methyl-androstane Derivatives. *J. Chem. Soc.* **1957**, 4105–4111. [↑](#endnote-ref-1)
